# Inhibitory effect of Fisetin against the aggregation process of SOD1 E100K mutant: computer-based drug design as a potential therapeutic for ALS disease

**DOI:** 10.3389/fchem.2025.1569777

**Published:** 2025-05-22

**Authors:** Fatemeh Sadat Seyedi Asl, Nasrin Malverdi, Fatemeh Sadat Ataei Kachouei, Fatemeh Zarei, Shamim Ghiabi, Payam Baziyar, Mohsen Nabi-Afjadi

**Affiliations:** ^1^ Medical School, Tehran University of Medical Sciences, Tehran, Iran; ^2^ Department of Cell and Molecular Biology and Microbiology, Faculty of Biological Science and Technology, University of Isfahan, Isfahan, Iran; ^3^ Department of Biology, Shiraz University, Shiraz, Iran; ^4^ Department of Biology, Islamic Azad University, Arsanjan, Iran; ^5^ Department of Medical Chemistry, Faculty of Pharmacy, Tehran Medical Sciences, Islamic Azad University, Tehran, Iran; ^6^ Department of Molecular and Cell Biology, Faculty of Basic Science, University of Mazandaran, Babolsar, Iran; ^7^ Department of Biochemistry, Faculty of Biological Sciences, University of Tarbiat Modares, Tehran, Iran

**Keywords:** ALS, E100K mutant, SOD1 aggregation, natural polyphenols, MD simulation, ADMET analysis

## Abstract

Protein misfolding and aggregation in superoxide dismutase 1 (SOD1) are linked to the neurodegenerative disease amyotrophic lateral sclerosis (ALS). SOD1 mutations have a significant role in the pathophysiology and fast behavior of protopathic proteins in ALS illness. The E100K mutation may be useful in uncovering the pathogenic mechanism of SOD1 associated with ALS. According to several studies, giving small molecule inhibitors made from polyphenolic flavonoid compounds may be a viable treatment strategy for neurological conditions. Using molecular docking and MD simulations, we have identified a potential flavonoid drug that may successfully inhibit SOD1’s amyloidogenic activity. Puerarin, Fisetin, and Peonidin provided intriguing pharmacological hints during the initial screening of flavonoids. The Fisetin-E100K complex had a larger residual energy contribution and substantial binding than other flavonoid compounds. The findings showed that, unlike other materials, Fisetin increased the structural stability, hydrophobicity, and flexibility of the mutant while reducing the amount of β-sheets. Furthermore, to distinguish aggregation in the mutant (unbound/bound) states, we displayed modifications in the free energy landscape (FEL). As a result, Fisetin was identified as having therapeutic potential against the E100K, which might make it a viable pharmacological option for the creation of inhibitors that lower the chance of ALS death.

## 1 Introduction

Human copper/zinc superoxide dismutase (hSOD1) gene mutations give rise to amyotrophic lateral sclerosis (ALS). Sporadic and familial ALS, account for 90% and 5%–10%, respectively. Defects in SOD1 account for 12%–23% of all FALS instances (Andersen, 2006; Wroe et al., 2008). Thus far, studies have documented over 220 SOD1 mutations (Chen et al., 2021). The dimeric structure of SOD1 along with the entity of association Cu/Zn ions, and intramolecular disulfide links result in stable conformation (Chattopadhyay et al., 2008; Ding and Dokholyan, 2008). Mutations linked to ALS primarily cause lower protein stability and an increased susceptibility to SOD1 aggregation (Wright et al., 2013). Reduced metal ion concentration (Farrokhzad et al., 2024), reduced intramolecular disulfide connections, misfolding, absence of post-translational modifications, decreased net negative charge (Abdullah Waheed et al., 2024), much higher propensity to dissociate the dimer interface (Faradonbeh et al., 2025), disruption of the hydrogen bond network as well as hydrophobic bonds (Ashkaran et al., 2024) are all characteristics of SOD1 mutations associated with ALS. In the end, these elements promote and accelerate the formation of SOD1 oligomers, increasing the tendency of dimers to split into monomers (Baziyar et al., 2022a; Baziyar et al., 2022b; Byström et al., 2010; Furukawa et al., 2008; Namadyan et al., 2023; Oztug Durer et al., 2009; Zaji et al., 2023). However, the pathogenesis mechanism of SOD1 in ALS is still unknown. A strong line of evidence indicates that the SOD1 mutations’ toxicity results from an increase in function rather than a reduction in the SOD1 detoxifying effect (Ding et al., 2012). Furthermore, various mutations produce aggregates and fibrils with distinct morphologies, which are attributed to differences in local residue assembly and protein dynamics (Huai and Zhang, 2019). Targeting the SOD1 protein would provide an understanding of the pathological mechanism of SOD1 in ALS. Other promising therapeutic strategies include interference related to aggregate formation and destabilization in SOD1. Among the mutations, the deleterious mutation E100K is most frequently observed in FALS patients, which causes the rapid progress of the sickness and ultimately leads to death (Prudencio et al., 2009). Recent studies have identified a sequence in β6 with high aggregation potential (100–106) that can cause severe alterations in the SOD1 structure (Castillo and Ventura, 2009). Hydrogen bonds are among the earliest bonds to respond to structural anomalies generated by varied protein mutations to preserve the structure of the protein (Alemasov et al., 2017; Khare and Dokholyan, 2006; Vogt et al., 1997). Another efficient method to promote protein aggregation could be neutralizing or lowering the charge of SOD1 which in turn induces ALS (Mavadat et al., 2023a; Mavadat et al., 2023b). Dropping the negative charge of SOD1 by substituting Glu to Lys causes an augmented level of protein aggregation. Taken together, the computational/experimental results support the theory that the negative charge shift modulates SOD1 aggregation, as previously reported (Salehi et al., 2015; Tompa et al., 2020). No surgical drug has been found to yet to treat this illness. A glutamatergic neurotransmission inhibitor called Riluzole is being widely utilized to reduce the pathophysiology of ALS. Life expectancy can only be increased by 60 days with this drug (Bucchia et al., 2015; Miller et al., 2003). Edaravone, an alternative medicine that postpones the development of the disease through dipping reactive oxygen species, was recently approved to effectively decrease the pathology of ALS-linked. Nevertheless, studies have shown that the consumption of medicinal doses of Edaravone can lead to adverse outcomes resulting in both illness advancement and unforeseen illnesses. (Cho and Shukla, 2020; Jackson et al., 2019). Hence, significant measures should be taken to create novel economical, and convenient medication agents to enhance survival time as well as lessen adverse effects. Recent studies have proposed natural polyphenolic flavonoids possess anti-aggregation capacity toward chief amyloidogenic proteins, including SOD1 (Freyssin et al., 2018; Henríquez et al., 2020; Limanaqi et al., 2020; Malik and Wiedau, 2020). Research has demonstrated that certain polyphenolic flavonoid composites can disrupt the creation of amyloid fibrils, decrease their aggregation, and inhibit oligomerization, although the precise method by which flavonoids imply their positive impact on amyloid deposition is still unclear (Baptista et al., 2014; Meng et al., 2010; Ono et al., 2004; Sato et al., 2013). To date, a vast set of medicinal molecules have been identified that have preventative or inhibitory properties against the aggregation of mutant SOD1 (Amporndanai et al., 2020; Chantadul et al., 2020; Ueda et al., 2017). Baicalein and Quercetin are other anti-amyloidogenic agents that inhibit SOD1aggregation (Bhatia et al., 2020). Additionally, research indicates that kaempferol and kaempferide may prevent intracellular SOD1 aggregates and modify pathogenic SOD1-mediated cell death (Srinivasan and Rajasekaran, 2018a). Computer simulations, commonly referred to as *in silico* analysis, have become an effective way to study disease-causing mutations and their effects on protein structure, as has been used previously (Nabi-Afjadi et al., 2025). Technological advances in computational chemistry have enabled researchers to skillfully study the dynamic interaction of a drug with its target protein (Noorbakhsh Varnosfaderani et al., 2023). With the help of a robust pipeline and sophisticated advanced software, researchers can now accurately model, accurately simulate various biomolecules, and characterize the complex interactions between proteins and small drug molecules. To date, several computational studies have been reported that have clearly demonstrated the divergent pathogenic behavior of misfolded proteins and their dynamic interactions with potent drug molecules (Bhardwaj et al., 2021; Doss et al., 2016; Kumar and Doss, 2016; Sharma et al., 2021). Bioactive molecules hinder proteins from aggregating or inhibiting them, which leads to the retrieval of biophysiochemical properties. Hence, this study aimed to explore the possible anti-aggregation properties of polyphenolic flavonoids on E100K SOD1 mutation to confirm their therapeutic efficacy. Hence, we conducted molecular dynamics (MD) simulations and molecular docking to comprehend the method more in-depth. While this assessment can offer valuable understanding in this area, the processes and variables responsible for the production of amyloid fibrils and protein aggregation are not entirely understood.

## 2 Materials and methods

### 2.1 Structure retrieval

Crystal structures of WT-SOD1 and E100K were searched using the Protein Data Bank (PDB) PDB ID: 1PU0 1.70Å resolution, which has been implemented as an ideal crystallographic template for the ongoing computational work on SOD1 (Workman, 2022). SOD1 protein dimer (A and B subunits) was considered for analysis. In order to reduce steric hindrance, the mutant structure was then geometrically optimized using the Yet Another Scientific Artificial Reality Application (YASARA) (http://www.yasara.org/) computer software (Land and Humble, 2018). In order to find a geometrically optimal E100K SOD1 structure, the steepest descent approach was used.

### 2.2 Ligand development

To obtain the SDF format of the chemical structure of flavonoids (ligands), the PubChem database—a rich source of chemical substances—was used. Molecular Orbital PACkage (MOPAC) and a semi-empirical AM1 Hamiltonian model were used to modify the ligand’s form (Dewar et al., 1985). With the improved ligands, docking studies were then conducted. Molegro Virtual Docker (MVD) version 4.0.2 was used to reduce molecular energies and store the optimized structures for future docking-based research (Thomsen and Christensen, 2006).

### 2.3 Molecular docking studies

MVD software was utilized to conduct molecular docking to ascertain the E100K mutant’s binding effectiveness with flavonoids and/or ligands. The search techniques included in this docking program are the MolDock SE modules and the MolDock optimizer (Zamani Amirzakaria et al., 2021). The MolDock optimizer was used as a search algorithm because of its high accuracy (Thomsen and Christensen, 2006). The optimal ligand binding site inside the protein was identified by docking the 3D structures of the ligands with the E100K mutant. Although the flavonoids’ unblocked torsional connections allowed for flexible docking from the ligand’s point of view, the mutant protein’s firmness was preserved throughout the docking process. Since the coverage region around residue 100 has been identified as a hotspot for protein aggregation, it was selected as the ligand-binding site. A grid box of 50 × 60 × 60 Å was created, with grid points spaced at 0.375 Å to create gird maps that included the remnants of active sites. Consequently, the docking outcomes were assessed using the MVD rerack rating method. Although this algorithm determines the optimal postures more precisely than the docking scoring function (Singh et al., 2016), it is computationally more valuable. For further study and, as a last option, PoseView visualization, the best-docked, thermodynamically stable protein-ligand complexes were chosen.

### 2.4 Molecular dynamics (MD) simulation

We compared the structure of WT-SOD1 with the E100K mutant using molecular dynamics (MD) simulations. In this work, stability and dynamics were assessed using a SOD1 dimer (chains A and B) with the ID code 1PU0. The initial conformations of WT-SOD1, the mutant, and the protein-ligand complex were built using GROMACS 2020.1, and the MD simulations for the protein-ligand structures were based on the outcomes of docking experiments using the CHARMM36 force field. A topology file was constructed using a simple point charge (SPC) water model for solvation. After that, the models were placed within a dodecahedron box, and the appropriate amounts of Na^+^ and Cl^˗^ ions were added to neutralize the system. The proper binding sites were occupied by the metal ions Cu^2+^ and Zn^2+^. To reduce the system’s energy usage, the steepest descent method was applied (Marquardt, 1963). The temperature and pressure were kept stable at 1.0 bar and 310 K by employing the Parrinello–Rahman barostat with a 2 ps time coupling constant and the v-rescale thermostat with a 1 ps time coupling constant. The default protonation states of the amino acids in GROMACS, corresponding to a pH of approximately 7.4 and standard pKa values, were used. The systems were then subjected to NVT equilibration at 310 K for up to 100 ps, followed by 100 ps of NPT equilibration at 1 bar with 1,000 kJ/mol restraint forces. Newton’s equation integration during the MD simulation was performed using the leap-frog algorithm (Van Gunsteren and Berendsen, 1988) with a time step of 2 fs, and data were recorded every 10 ps. The protein’s covalent bonds were constrained to maintain constant bond lengths using LINCS (Hess et al., 1997). The Particle Mesh Ewald (PME) approach was used to calculate electrostatic interactions (Darden et al., 1999). All atoms were free to move throughout MD simulations of WT-SOD1, the mutant, and the protein-ligand complexes at 310 K for 250 ns. GROMACS tools, including gmx rms, gmx rmsf, gmx gyrate, gmx h-bond, and gmx dssp, were used to examine the generated trajectories. These tools provided parameters such as radius of gyration (Rg), secondary structure (SS), hydrogen bonds (H-bond), root-mean-square deviation (RMSD), and root-mean-square fluctuation (RMSF). Additionally, the MD trajectories were analyzed using the Bio3D library in R software (Grant et al., 2006), where principal component analysis (PCA) was performed on the WT-SOD1 and its variants. The PCA focused on the Cα atoms of the protein, with covariance matrices generated from their Cartesian coordinates.

### 2.5 MM-PBSA calculation

One crucial stage in an *in silico* drug design process that establishes the binding affinity of inhibitors to the receptor is the binding free energy. MM/PBSA, which specifies the free energy of protein and ligand binding as follows, was used to calculate the free energy of binding for ligand-receptor complexes according to the following equation (Equation 1):

$$\Delta G_{\text{binding}}=\Delta G_{\text{complex}}\;–\;\left(\Delta G_{\text{protein}}+\Delta G_{\text{ligand}}\right)$$ (1)

The protein-ligand complex’s overall MMPBSA energy is shown by ΔG_complex_, whereas the solution free energies of the individual proteins and ligands are shown by ΔGprotein and ΔGligand, respectively. The following is an expression for the free energy of each individual’s existence according to the following equation (Equation 2):

$$\Delta G=E_{\text{MM}}+G_{\text{solvation}}\;‐\text{ TS}$$ (2)

G_solvation_ specifies the free energy of solvation, while E_MM_ displays the average molecular mechanical potential energy in the vacuum. While T and S describe the temperature and entropy, respectively, TS together illustrates the entropic contribution to the free energy in vacuum. The bond angle, torsion, electrostatic (E_elec_) and van der Waals (E_vdw_) interactions, as well as other bonded and nonbonded interactions of the molecules, are also included in the E_MM_. Finally, both electrostatic and nonelectrostatic (G_polar_ and G_nonpolar_) components are present in the free energy of solvation and G_solvation_ (Kumari et al., 2014).

### 2.6 Free energy landscape

By employing the conformational sampling approach, the free energy landscape of the protein was identified, resulting in the almost natural structural conformation. Here, flavonoid conformational sampling was utilized to build the free energy landscape (FEL) for mutant SOD1 and its complex using simulated trajectories. Thus, the FEL was built using the following equation (Equation 3), which uses RMSD and Rg trajectories in addition to free energy:

$$\Delta G\;\left(p1,p2\right)=‐k_{B}T\ln\rho \left(p1,p\;2\right)$$ (3)

In this case, ΔG stands for the state’s Gibbs free energy, kB for the Boltzmann constant, and T for the simulation’s temperature. The joint probability distribution ρ (p1, p2) represents the reaction coordinates of the FEL, *p*1 and *p*2 (Papaleo et al., 2009).

### 2.7 Cross-correlation matrix

Fluctuations of protein alpha carbon atoms during the dynamic period via the dynamic cross-correlation matrix (DCCM) DCCij were calculated using g_covara to evaluate the collective motions of the WT-SOD1, E100K mutant, and mutant-ligand complexes, respectively. A covariance matrix was constructed between *j* and *i* atoms, which measures the correlated nature of atomic fluctuations. The correlation calculation equation is as follows (Equation 4):

$$\text{DCC }\left(i,j\right)=\left(\Delta r_{i}\;x\;\Delta r_{j}\right)\;/\;\sqrt{\Delta r_{i}^{2}}\;x\;\;\Delta r_{j}^{2}$$ (4)

Herein, Δri and Δrj pertain to the vectors of atomic displacement for atoms i and j from their average position during stipulated time intervals (McCammon, 1984).

### 2.8 Drug metabolism and pharmacokinetics

Additionally, pkCSM (http://biosig.unimelb.edu.au/pkcsm/prediction) was utilized to evaluate the toxicity of the compounds profiled using the Shimadzu GC-MS-QP 2010 Ultra (Pires et al., 2015). The canonical SMILES for the molecular structure of each chemical were obtained from PubChem (https://pub-chem.ncbi.nlm.nih.gov). The compounds with the requisite physicochemical properties were subsequently investigated for their pharmacokinetic properties.

## 3 Results and discussion

### 3.1 The substitution mutation modifies the β-barrel stability and surface electrostatic potential of SOD1

**FIGURE 1:**
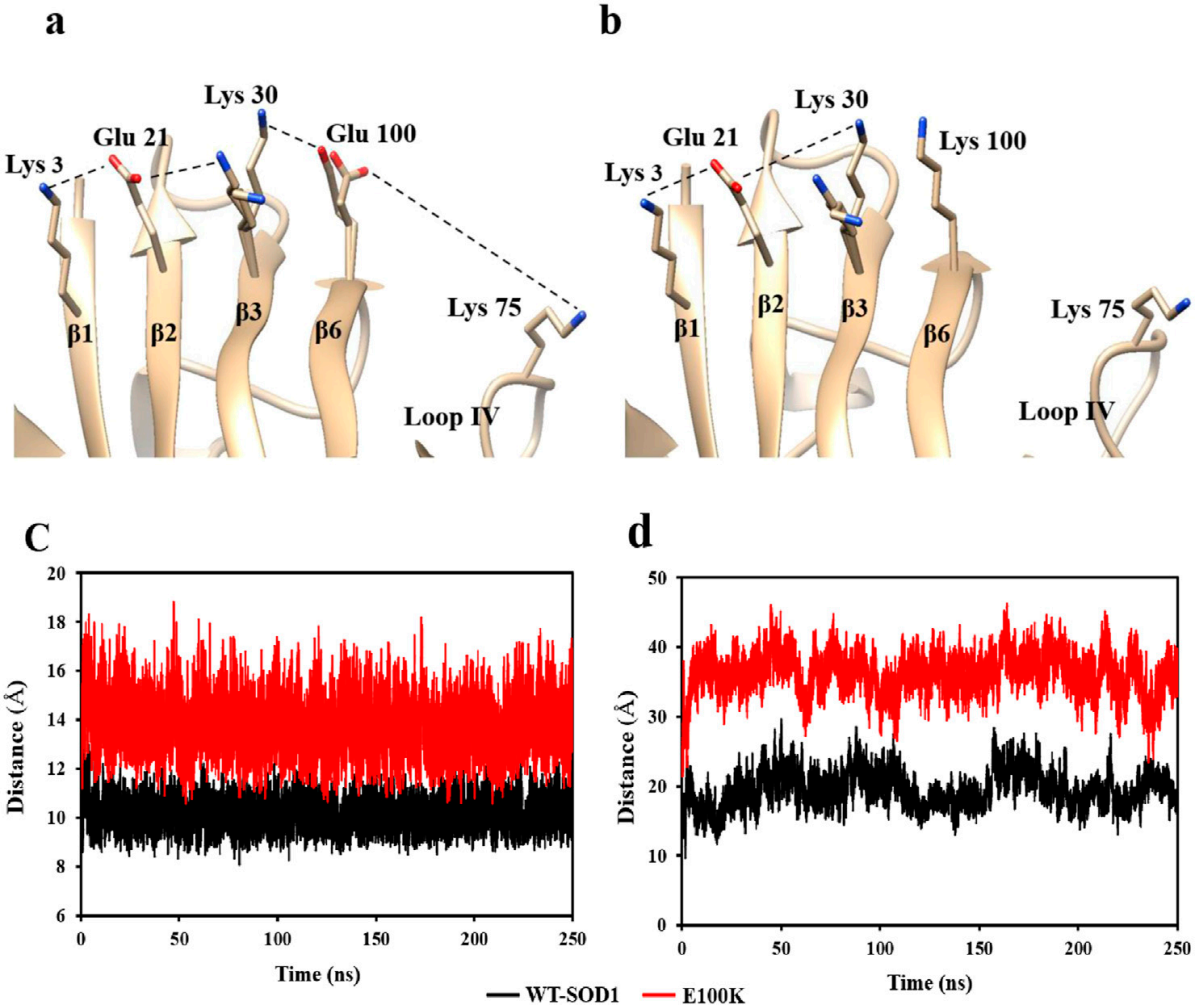
Diagram illustrating the chain of ionic interactions formed on the outside of the β-strands by Lys3 (β1), Glu21 (β2), Lys30 (β3), and Glu100 (β6), which support the stability of the wild-type SOD1 **(a)** and the E100K mutant **(b)**. The diagram shows that Lys replaces Glu100, causing two Lys residues (k100 and k30) to be placed next to each other concurrently. The PDB entry 1PU0 and the Chimera program were used to create the figure. The changes in the distance between the mutant SOD1E100K (red line) and the wild-type SOD1 (black line) between **(c)** Glu100-Lys30 and **(d)** Glu100-Lys75 during the simulation.

Protein misfolding has been seen as a result of β-6 strand mutations, which can interfere with the hydrophobic core of the β-barrel’s packing. The results of mutations imply that modifications in secondary structures, hydrophobicity, flexibility, stability, and protein folding ability can all be influenced by conformational shifts and local interactions. By neutralizing the positive charges, Glu100, which is present on the outer surface of the β-barrel (β6) in WT-SOD1, preserves the SOD1 surface electrostatic potential. The β-barrel stability and surface electrostatic potential of SOD1 are altered by a mutation at position E100. The net negative charge in the protein’s plane decreases when Glu100 is substituted for Lys, and this can boost aberrant contacts for nuclear aggregation (Prudencio et al., 2009; Sandelin et al., 2007). The E100K mutant was simulated using Chimera software, and the results indicate that the substitution of Lys for Glu100 eliminates hydrogen bonding and electrostatic interactions, resulting in the simultaneous placement of three Lys residues side by side (Figures 1a,b). Additionally, the repulsive force or spatial interferences between side chains may cause the protein to partially unfold and reveal hydrophobic patches. The replacement of this particular residue in Glu100 alters the protein’s net charge and impacts the β-barrel’s stability by influencing the chain of native electrostatic interactions with the charged residues Lys30, Glu21, and Lys3, which are situated in the adjacent strands of β3, β2, and β1, respectively (Parge et al., 1992). Throughout the simulation, the β6 and β3 strands’ electrostatic attraction is eliminated following the mutation. To be more precise, in the WT protein, the distance between Glu100 (β6) and Lys30 (β3) was 10.14 Å, while in the E100K protein, it rose to 13.7 Å (Figure 1c). The stability of the β-barrel may be affected and other interactions in the charging network may be impacted by this modification in the native contact between Glu100-Lys30. Overall, our findings suggested that mutations neutralizing or decreasing the protein’s net negative charge would be important in decreasing protein stability, which might result in misfolding and shorten the lag phase in the SOD1 aggregation process (Sandelin et al., 2007).

### 3.2 Increased conformational disorder in loop IV/metal-binding loop by mutation at Glu100

Electrostatic connections of Glu100 (β6)-Lys75 and Asp101(β6)-Arg79, connecting Lys75 and Arg79 present in loop IV/metal-binding loop with the β-barrel residues, greatly maintain the conformation of loop IV in WT-SOD1. Interestingly, the lack of stabilizer contractions with the β-barrel results in enhanced flexibility in loop IV in our molecular dynamics simulations of E100K. According to the current MD data, the mutations at Glu100 cause Lys75 and Lys100 to be further apart, leaving loop IV in a relaxed conformation. The E100K mutant exhibited a considerably larger average distance (36.2 Å) between Lys75 and Lys100 when compared to the WT-SOD1 (19.5 Å for Lys75 and Glu100). This difference may be attributed to electrostatic repulsion, which causes loop IV to obtain distance from the β-barrel (Figure 1d). Likewise, Asp101 (at the β6 end, next to Glu100) and Arg79 of loop IV lose their hydrogen bond as a result of the mutation. The E100K mutant’s notable increase in distance might perhaps be attributed to the reorientation of Arg79’s side chain from its initial conformation. In addition, the Arg79 side chain’s changed conformation breaks its H-bonds with Pro74 and Asp83, zinc, and loses its connections with Asp101. These modifications aid in preserving the IV ring’s proper fold at its original location. In WT-SOD1, the side chain of Arg79 forms hydrogen bonds with the carbonyl oxygen of Pro74 and the side chain of Asp101, connecting the loop region on loop IV to the β-barrel. As a result, loop IV’s structural instability is increased in Glu100 mutants due to the lack of these stabilizing connections. Because native stabilizing connections between loop IV and the β-barrel are lost, mutation at Glu100 modifies the superficial electrostatic potential of SOD1 and improves loop IV’s flexibility. These results are consistent with those previously reported (Tompa et al., 2020).

### 3.3 ALS-associated SOD1 mutations and subsequently therapeutic effects of flavonoids

ALS mutations in SOD1 have been thoroughly studied, and the results have demonstrated that these mutations heighten the protein’s inclination to aggregate. It should be highlighted, nonetheless, that mutations that cause fALS do not necessarily result in a rise in SOD1’s aggregation propensity or a reduction in its lag time. Even when several mutations target the same amino acid position, the intrinsic propensity of distinct mutants to produce aggregated protein differs. The length of the disease is correlated with the inherent aggregation tendency of ALS mutations; hence, mutations with a strong aggregation propensity are linked to a higher risk and a shorter disease duration (Prudencio et al., 2009). In the past, several therapeutic approaches have been put forth to treat neurological illnesses, including stopping protein self-assembly, eliminating amyloid masses that have already formed, decreasing the creation of misfolded protein types by stabilizing the native state of the protein, changing toxic oligomeric species into non-toxic species, etc. Several tiny compounds have been found to have therapeutic promise and may perform as probes to comprehend the molecular processes behind the production of amyloid (Cohen and Kelly, 2003; Härd and Lendel, 2012; Ladiwala et al., 2011; Sharma et al., 2010; Singh et al., 2013). Numerous natural medicinal substances have recently been suggested as treatments for many crippling neurodegenerative illnesses (Li et al., 2014). The pathogenic idea states that the main cause of neurodegenerative diseases is cytotoxic fibril aggregates in various brain tissues, hence new treatment agents that control and stop protein aggregation are always needed (Matsumoto et al., 2005). In particular, interest in natural polyphenolic flavonoids has increased due to their capacity to create chemical interactions (Ferreira et al., 2011; Singh et al., 2014). The ability of flavonoids to effectively alter the unique dynamic pathogenicity status of mutants has recently been validated by a number of computational publications (Srinivasan and Rajasekaran, 2016; Srinivasan and Rajasekaran, 2017; Srinivasan and Rajasekaran, 2018b). Since flavonoids control, inhibit, and lessen the cytotoxic character of pathogenic SOD1, current experimental investigations provide compelling scientific evidence for their usage against aberrant SOD1 masses (Ueda et al., 2017). Since ALS significantly reduces life expectancy, it is recognized as the most deadly neurological disorder (Rowland and Shneider, 2001). In a similar vein, flavonoids such as Peonidin, Fisetin, and Puerarin have been proposed to have anti-amyloidogenic qualities in the face of severe illnesses including Parkinson’s, Alzheimer’s (Asl et al., 2025), and ATTR amyloidosis (Li et al., 2022; Sawmiller et al., 2016), which may function as an anti-pathogenic SOD1 analeptic (Abdullah Waheed et al., 2024; Noorbakhsh Varnosfaderani et al., 2023; Qassim et al., 2023). Type 2 diabetes (Sharbatdar et al., 2023), antioxidant (Naghmachi et al., 2022), antimutagenic, anti-cancer (Li et al., 2023), oxidative (Khakpour et al., 2024), apoptotic markers, anti-inflammatory, and anti-carcinogenic activity (Abotaleb et al., 2018) are some of the other beneficial qualities that polyphenolic flavonoids offer. In light of the scientific harm caused by the E100K mutant in ALS and its aggregation-prone properties, the anti-aggregation properties of natural polyphenols were the primary focus using computational analysis as a guide in this study.

### 3.4 Binding interaction analysis

Molecular docking creates and rates receptor-ligand positions based on their interaction energies. Molecular docking is considered a vital approach to characterizing protein-ligand affinity (Thirumal Kumar et al., 2017). After a comprehensive literature review, 23 polyphenols were recognized as promising flavonoid candidates for computational assessment of amyloidogenicity on E100K, which had inhibition or/and sanative effects on amyloid fibril masses. These compounds included: Puerarin, Fisetin, Peonidin, Malvidin, Delphinidin, Diosmetin, Hesperetin, Pelargonidin, Quercetin, Nobiletin, Glycitein, Tangeretin, Luteolin, Pratensein, Epicatechin, Daidzein, Apigenin, Kaempferol, Naringenin, Genistein, Chrysin, Baicalein and Galangin (Li et al., 2022). During the primary testing, docking was conducted on polyphenolic flavonoids with E100K. The mean docking score values ​​of the flavonoid bonds between the A and B chains of SOD1 are presented in Table 1.

**TABLE 1:** Calculated docking score of flavonoids with E100K mutant SOD1 protein. These values ​​are obtained from the average of two SOD1 chains.

S.no	Polyphenols	Docking score
1 2 3 4 5 6 7 8 9 10 11 12 13 14 15 16 17 18 19 20 21 22 23	Puerarin Fisetin Peonidin Malvidin Delphinidin Diosmetin Hesperetin Pelargonidin Quercetin Nobiletin Glycitein Tangeretin Luteolin Pratensein Epicatechin Daidzein Apigenin Kaempferol Naringenin Genistein Chrysin Baicalein Galangin	−93.2529 −90.046 −85.8619 −85.5701 −83.7969 −81.7649 −79.5395 −78.7266 −76.8227 −76.7522 −76.5791 −75.2496 −74.7731 −74.0508 −73.8354 −73.652 −73.5498 −72.8585 −72.0946 −71.2376 −70.2073 −68.6243 −67.9087

It has been revealed when the drug/ligand forms a strong binding with its target, the induced effect is superior (Sneha and Doss, 2016). The binding free energy determines quantified interactions between the ligand and its target. Based on this parameter, Puerarin, Fisetin, and Peonidin were shortlisted as promising compounds for further analysis. It was illustrated that Puerarin possesses the maximum binding efficiency with E100K mutant protein, followed by Fisetin and Peonidin. The binding positions for every representative mutant SOD1 compound from cluster analysis in 3-D diagrams are as follows.

#### 3.4.1 Chain-A interactions

**FIGURE 2:**
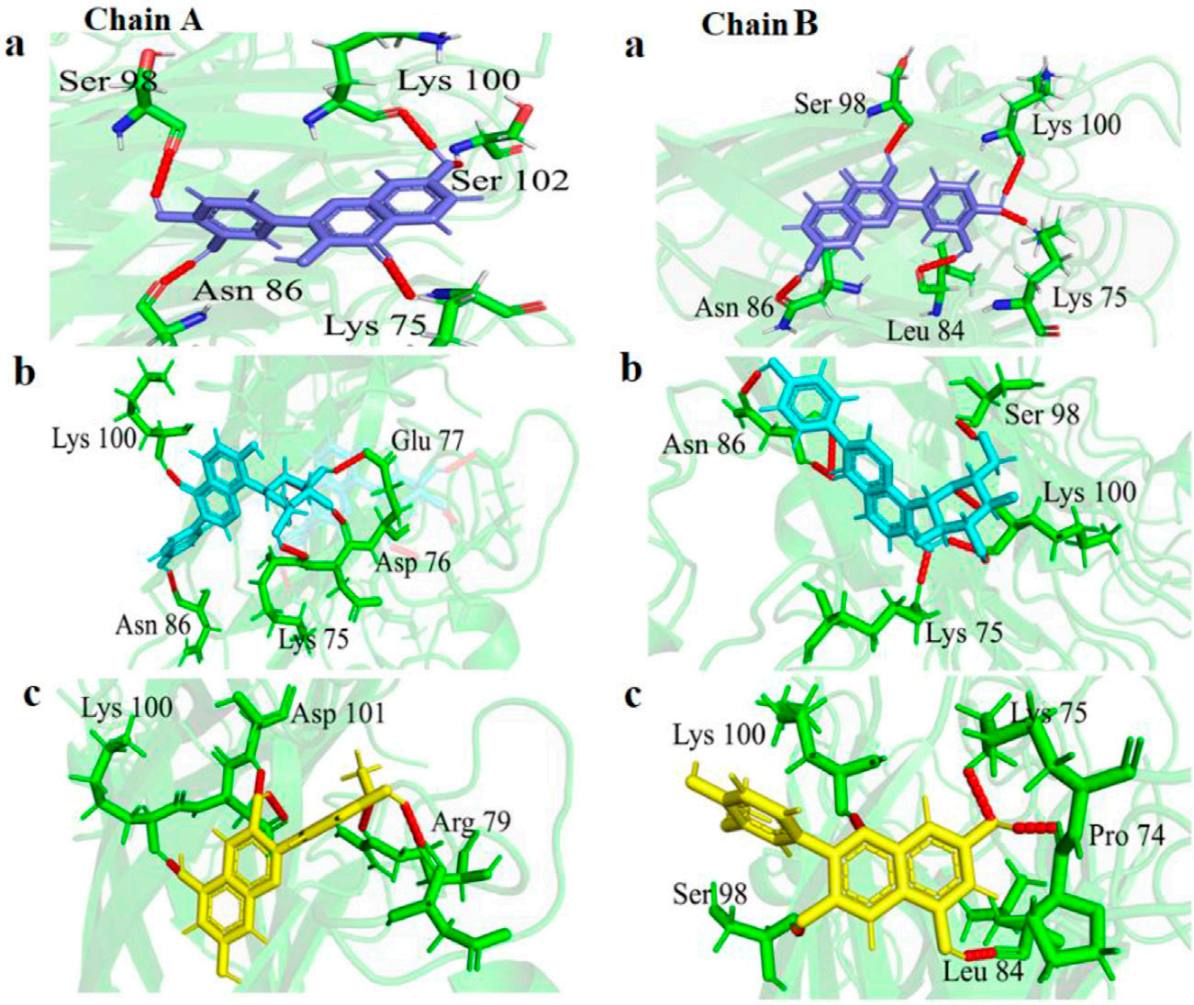
Visualization of variation in 3-D protein-ligand interactions between E100K mutant docked with **(a)** Fisetin, **(b)** Puerarin, and **(c)** Peonidin. Hydrogen bonds are depicted by dashed red lines.

In chain-A, Fisetin formed hydrogen bond with residues Lys75, Asn86, Ser98, Lys100 and Ser102 (Figure 2a). Puerarin formed H-bond with Lys75, Asp76, Glu77, Asn86 and Lys100 (Figure 2b). Peonidin formed H-bond with Arg79, Lys100 and Asp101 (Figure 2c).

#### 3.4.2 Chain-B interactions

In chain-B, Fisetin formed hydrogen bond with residues Lys75, Leu84, Asn86, Ser98 and Lys100 (Figure 2a). Puerarin formed H-bond with Lys75, Asn86, Ser98 and Lys100 (Figure 2b). Peonidin formed H-bond with Pro74, Lys75, Leu84, Ser98 and Lys100 (Figure 2c).

**FIGURE 3:**
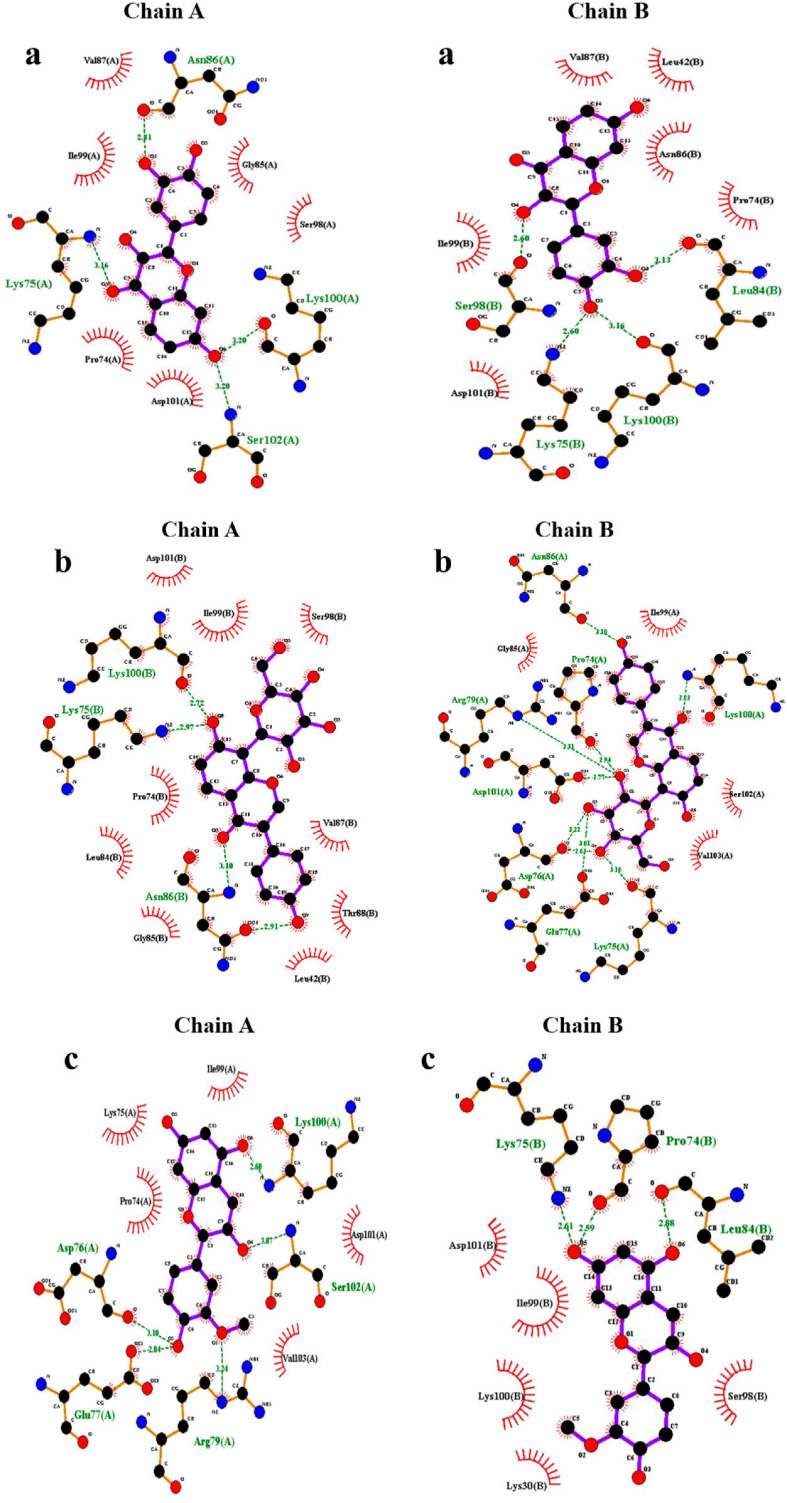
Illustration of discrepancy in 2-D protein-ligand connections between E100K mutant of SOD1 enzyme docked with **(a)** Fisetin, **(b)** Puerarin, and **(c)** Peonidin. Hydrogen and hydrophobic bindings are created by flavonoids.

The altered orientation and molecular size of Fisetin (creating five hydrogen interactions in both chains) in the mutant augmented the hydrogen interaction network in comparison to other flavonoids. Furthermore, the binding sites of each flavonoid to the E100K mutant in 2-D diagrams were examined (Figure 3). It is notable that intermolecular interactions have a dynamic impact on the binding affinities between proteins and ligands and are not only dependent on hydrogen bond interactions to maintain the binding affinities (Patil et al., 2010).

Additionally, as shown in Table 2, phenyl rings at the polyphenolic flavonoids were essential in forming hydrophobic contacts with mutant-ligand complexes SOD1. The ligand-protein interaction affinity increased upon a surge in hydrophobic interactions. As the hydrophobic interactions within the protein-ligand complex’s active region grew, the biological activity also increased. As a result, complementary docking studies revealed a strong interaction between flavonoids and mutant SOD1, indicating their potential in preventing or inhibiting the aggregation tendency of SOD1.

**TABLE 2:** Interactions formed by mutant SOD1 binding residues with protein–ligand complexes.

Chains	Ligands interacting with mutant SOD1	Residues involving binding interactions
		Hydrophobic interaction
A	E100K-Fisetin E100K-Puerarin E100K-Peonidin	Pro74, Gly85, Ser98, Ile99, Val87 and Asp101 Leu42, Pro74, Leu84, Val87, Thr88, Gly85, Ser98, Ile99 and Asp101 Pro74, Lys75, Ile99, Asp101 and Val103
B	E100K-Fisetin E100K-Puerarin E100K-Peonidin	Leu42, Pro74, Asn86, Val87, Ile99 and Asp101 Gly85, Ile99, Ser102 and Val103 Lys30, Ser98, Ile99, Lys100 and Asp101

### 3.5 Molecular mechanics Poisson Boltzmann surface area calculations

MM-PBSA is an acknowledged, effective, and accurate technique for portraying protein-ligand binding affinities (Kumari et al., 2014). MM-PBSA strategy to estimate binding free energy has become one of the methods used to calculate interaction energies (Bhardwaj and Purohit, 2021). To validate the accuracy of the binding energies obtained from molecular docking investigations, the ΔE_bind_ of ligand-mutated complexes was calculated using the MM-PBSA approach. Binding free energy and components acquired from MM-PBSA computations of designated protein-ligand complexes are exposed in Table 3. In chain-A, the binding free energy revealed by Fisetin, Puerarin, and Peonidin these values were −25.11 ± 2.37, −17.61 ± 1.64, and −12.99 ± 2.58 kcal/mol, respectively, while in B-chain, the binding free energies revealed by Fisetin, Puerarin, and Peonidin were −22.28 ± 2.47, −18.26 ± 2.36 and −10.31 ± 1.46 kcal/mol, respectively. The Van der Waals, electrostatic energy, ΔE (ENPOLAR), and ΔGGAS positively affected the overall free energy of binding in every nominated complex, except for the electrostatic energy of Peonidin. On the other hand, the solvation energy negatively affected the overall free energy of binding in every nominated complex. Fisetin showed a stronger affinity with the E100K mutant compared to other flavonoid compounds.

**TABLE 3:** The mutant complexes’ average binding free energy (MM/PBSA) values. The unit of measurement for all energies is kcal/mol.

Chains	Complexes	ΔVDW	ΔEEL	ΔEPB	ΔGgas	ΔGsolv	ΔE_bind_	ΔE(ENPOLAR)
A	E100K-Fisetin	−20.4	−25.86	31.35	−46.26	29.01	−25.11	−2.34
	E100K-Puerarin	−34.47	−33.91	48.06	−68.38	44.6	−17.61	−3.46
	E100K-Peonidin	−21.07	−9.58	17.63	−30.65	15.42	−12.99	−2.21
B	E100K-Fisetin	−23.26	−18.62	30.12	−41.87	27.56	−22.28	−2.56
	E100K-Puerarin	−28.5	−18.89	30.87	−47.39	27.74	−18.26	−3.13
	E100K-Peonidin	−15.82	4.44	3.14	−11.38	1.47	−10.31	−1.67

VDW (van der Waals energy), EEL (electrostatic energy), EPB , the electrostatic contribution to the solvation-free energy calculated by PB (MMPBSA, polar solvation energy), ΔGGAS (gas-phase molecular mechanics free energy), ΔGSOLV (solvation free energy), ΔG TOTAL (net system energy) = ΔE_bind_, binding free energy, ΔE (ENPOLAR): Nonpolar contribution of repulsive solute-solvent interactions to the solvation energy.

**FIGURE 4:**
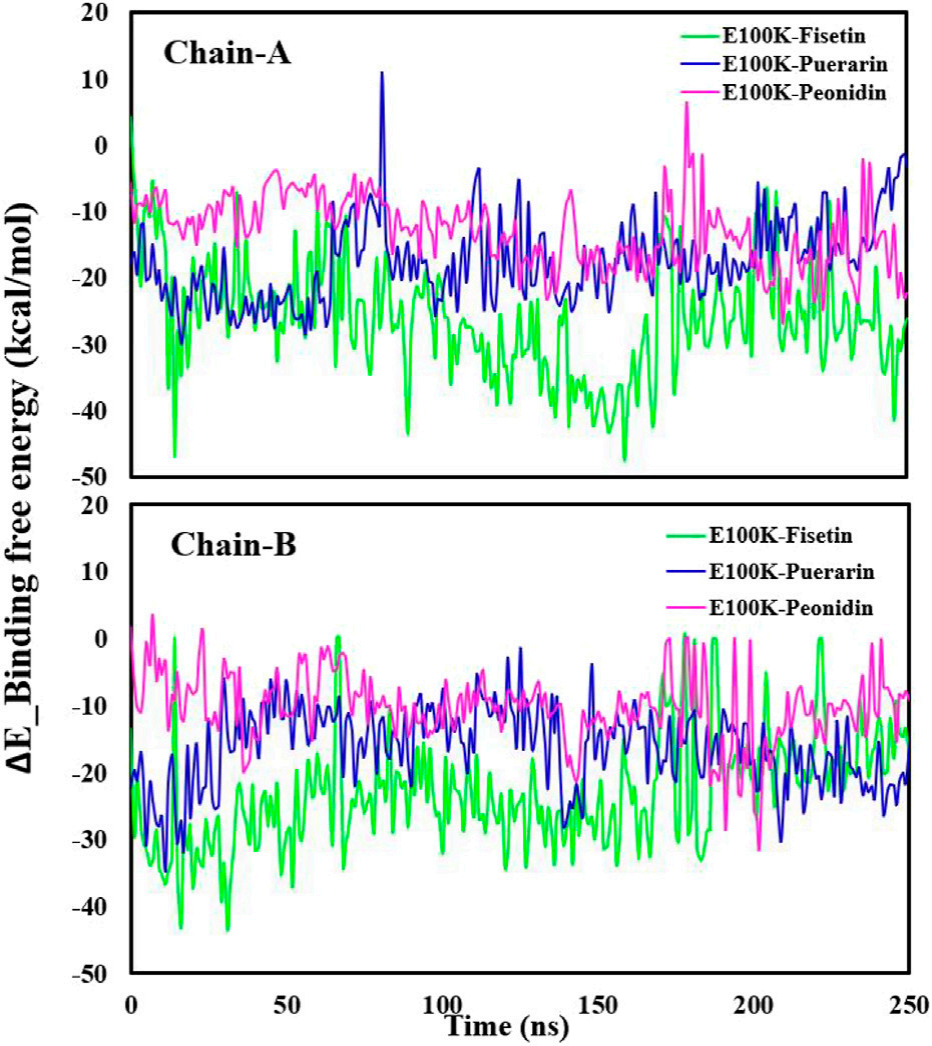
Illustration of the Delta_E_Binding free energy kcal/mol of E100K with Puerarin (Blue), Fisetin (Green), and Peonidin (Purple). The data were expressed as the mean ± SD (n = 3).

**FIGURE 5:**
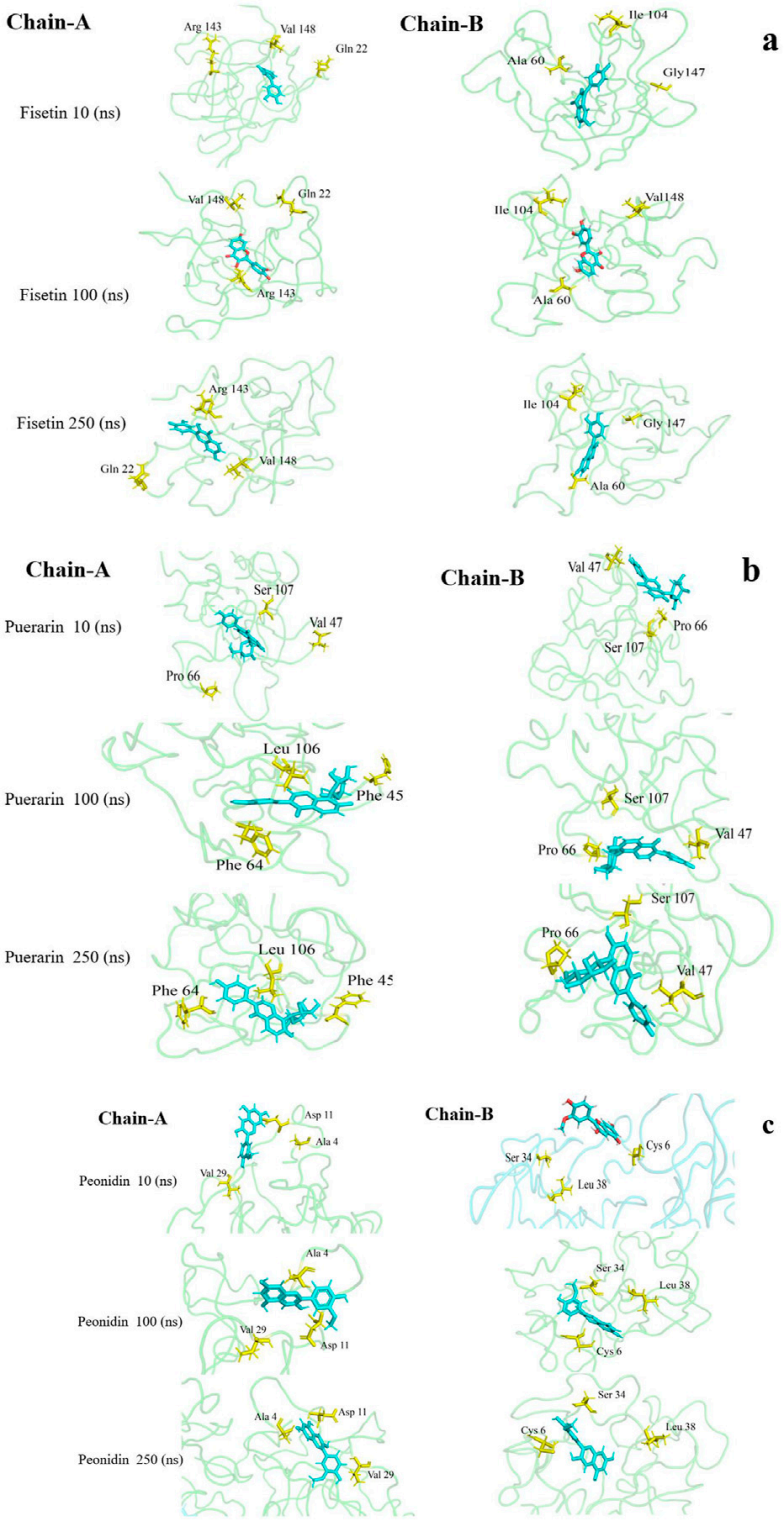
Visualization of variation in 3D protein-ligand interactions (changes in their initial binding positions during simulation) between the bound E100K mutant docked with **(a)** Fisetin, **(b)** Puerarin, and **(c)** Peonidin.

Figure 4 illustrates average binding free energy diagrams for both chains. The results confirmed previous observations of molecular binding and showed that the selected Fisetin molecule binds more efficiently with E100K mutant than other flavonoid compounds. The affinity of the molecules is positively correlated with the molecules’ ability to avert or inhibit aggregation due to the E100K mutation. Hereafter, we recommend that Fisetin can act better than other flavonoid compounds in treating ALS by reducing the influence of polymorphisms on conformational changes that inhibit aggregation. According to the present results, it is evident that structural changes occur during the simulation period. To assess whether these motions can affect ligand binding, changes in their initial binding positions along the trajectories are shown in Figure 5. The results show that Fisetin forms the most stable bonds with the residues compared to other compounds.

### 3.6 MD simulation analysis of protein–ligand complexes

Despite the valuable information about protein-ligand interaction obtained from molecular docking, it has limited practice in drug development due to the predicted static positions. Naturally, protein-ligand interplays have a dynamic nature; therefore, MD simulation is widely used in the field of computational biology to interpret the binding/unbinding procedure of ligands from several bio-molecules. MD simulation helps to confirm the molecular binding created by protein-ligand interplays and evaluate the therapeutic progress of molecules in drug development. Therefore, the structural dynamics of WT-SOD1 and mutant E100K protein and also the interaction of flavonoids (selected bioactive molecules) as mutant-ligand complex was investigated by MD simulation for 250 ns at 310 K. Upon simulation, analysis of trajectories provided a unique outlook on the active motions of the mutant and its flavonoid-based compounds. In the present study, all systems underwent three rounds of 250 ns of simulations. Data were expressed as mean ± standard division (SD), (n = 3).

**FIGURE 6:**
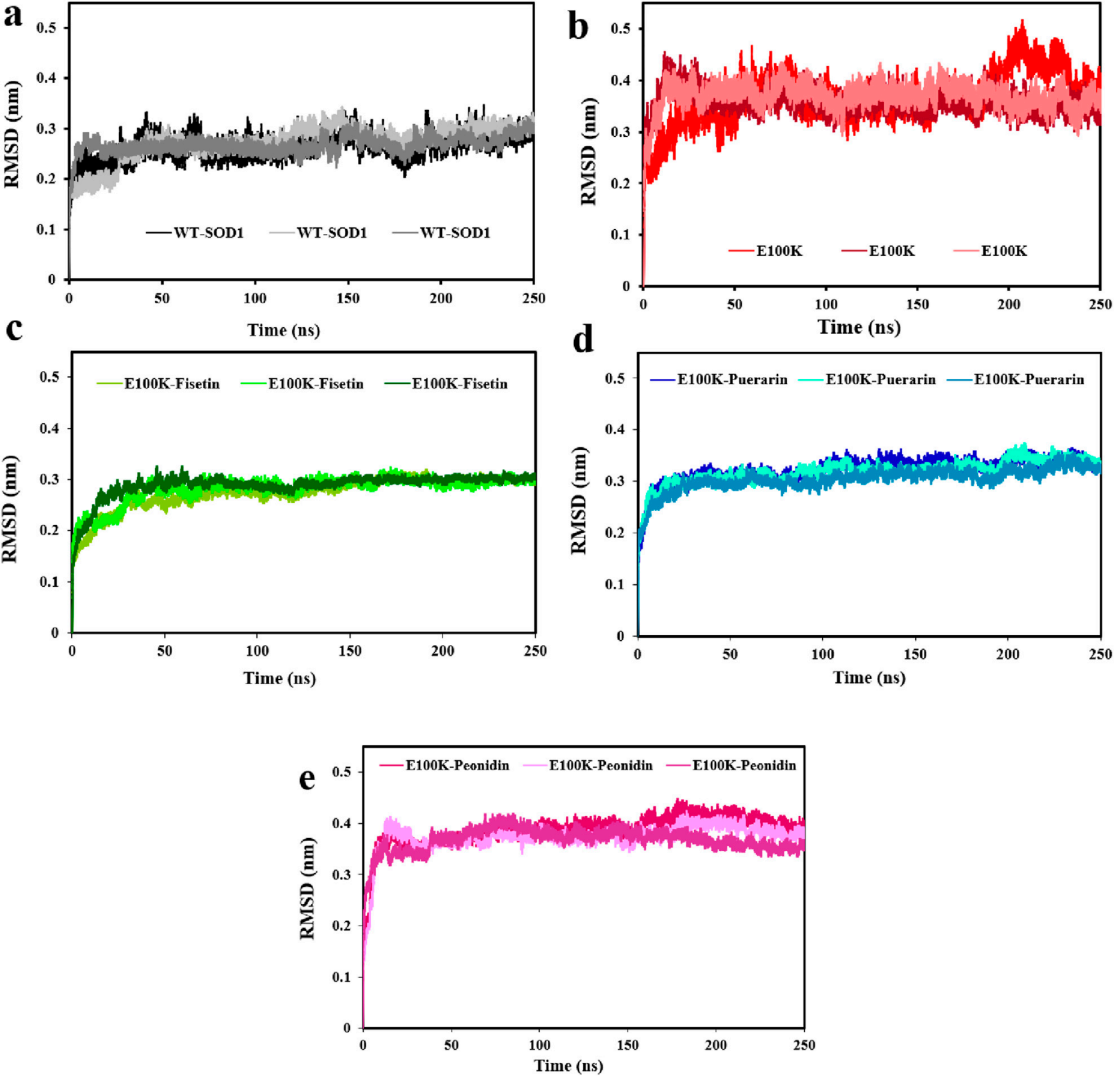
Changes in WT-SOD1 and E100K mutant root-mean-square deviation (RMSD), **(a)** WT-SOD1, **(b)** E100K upon interacting with flavonoids: **(c)** Fisetin, **(d)** Puerarin, and **(e)** Peonidin. RMSD values ​​ were performed from three independent trajectories for each WT, mutant, and protein-ligand complexes. Data were expressed as mean ± SD (n = 3).

The steadiness of a protein considering its conformational structure may be assessed using deviation during protein simulation. Therefore, in the first phase, the variance of a protein’s carbon-alpha atoms’ original structural composition and final location, measured as the root mean square deviation (RMSD), was employed (Jiang et al., 2019). To determine the steadiness of the protein-ligand complexes, the RMSD value for the Cα backbone was monitored by examining the MD trajectories every 20 ns. According to the results, the RMSD values averaged 0.268 ± 0.025 nm for WT (Figure 6a), and 0.365 ± 0.031 nm for E100K mutant (Figure 6b). The general trend of the results showed that the mutation will lead to a distinct protein composition, which indirectly indicates a loss of protein stability compared to the WT. Interaction of Fisetin with E100K decrease RMSD to 0.282 ± 0.027 nm (Figure 6c). However, the interaction of Puerarin with the E100K mutant resulted in a decrease in RMSD to 0.315 ± 0.024 nm (Figure 6d). Nonetheless, after the interaction of Peonidin with the E100K mutant, the RMSD value increased to 0.376 ± 0.028 nm (Figure 6e). These results were promising that the binding of polyphenolic phytochemical compound (Fisetin) to E100K might raise the structural resemblance between WT and E100K compared to other compounds. Overall, the calculated RMSD supports the simulated results of the protein–ligand complex leading to a stable pathway, which will ultimately help further analyses.

**FIGURE 7:**
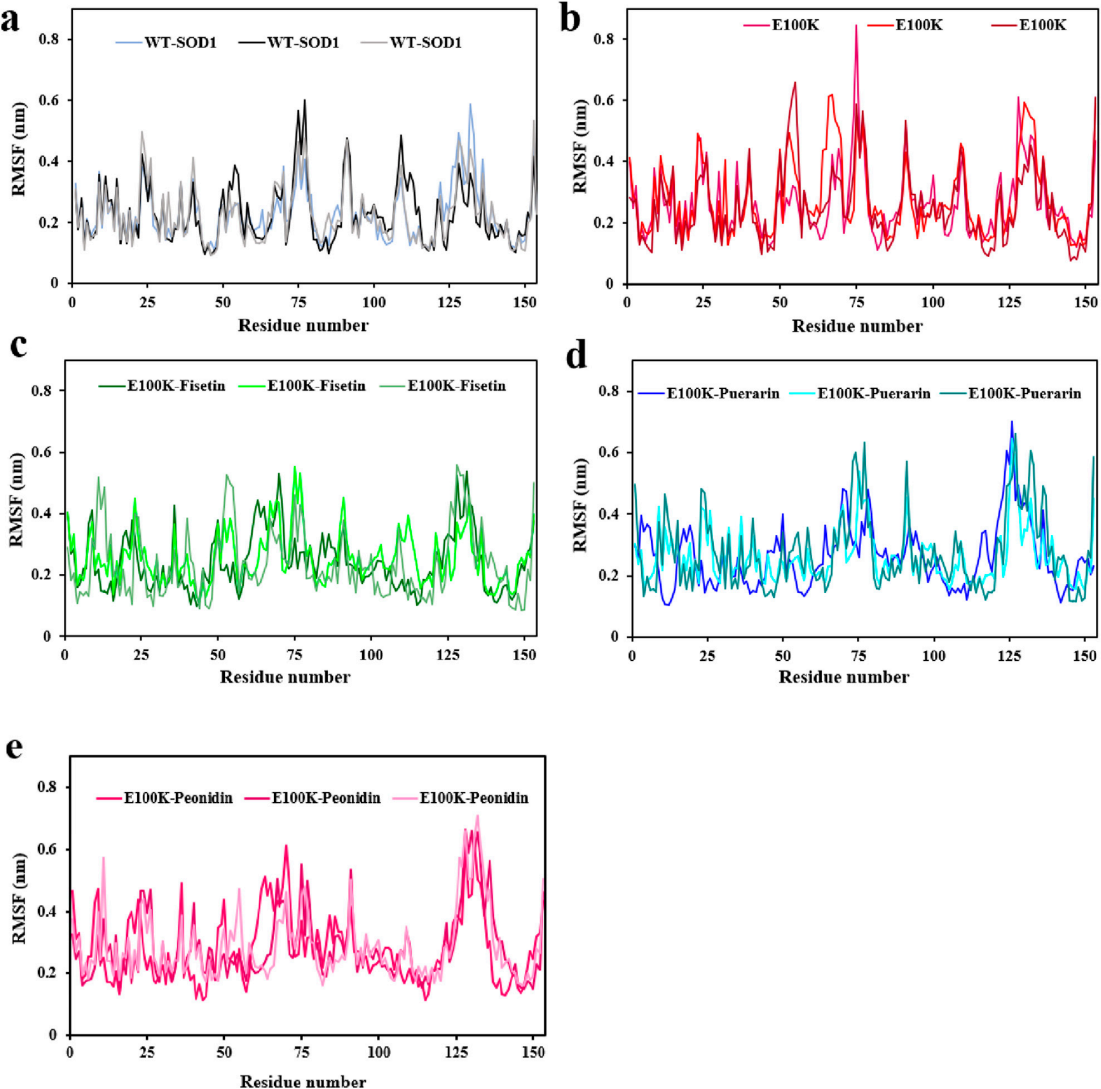
Molecular dynamics (MD) simulations-based root-mean-square fluctuation (RMSF) of the backbone Cα-atoms of the **(a)** WT-SOD1, **(b)** E100K upon interacting with flavonoids: **(c)** Fisetin, **(d)** Puerarin, and **(e)** Peonidin. RMSF values ​​ were performed from three independent trajectories for each WT, mutant and protein-ligand complexes. Data were expressed as mean ± SD (n = 3).

To assess the mobility of structural elements, RMSD was deemed insufficient, therefore, the root-mean-square fluctuations (RMSF) for the Cα atoms of the WT, mutant, and protein-ligand complexes were employed to investigate the flexibility of the structure. RMSF is a main factor that calculates the deviation of a group of atoms in a structural system through MD simulation. (Jiang et al., 2019). Hence, we conservatively decomposed the flexibility values for each remaining section to inspect the structural stability feature. On average, the flexibility values in both chains for the WT-SOD1 (Figure 7a), and E100K mutant (Figure 7b) were 0.228 ± 0.09 and 0.264 ± 0.11 nm, respectively. Remarkably, the mean residual flexibility decreased to 0.245 ± 0.11 nm upon interaction with Fisetin (Figure 7c). The interaction with Puerarin did not change much in the mean residual flexibility and remained approximately the same at 0.267 ± 0.1 nm (Figure 7d). But when Peonidin was complexed with the E100K mutant, the average residual flexibility increased to 0.285 ± 0.11 nm (Figure 7e). Relative to the WT, E100K showed higher flexibility among residues forming the functionally significant loops including loop IV/metal-binding loop (residues 49–83) and loop VII/electrostatic loop (residues 121–142), and a decrease in flexibility was observed in the residues creating β-sheets. Generally, the loops showed greater fluctuations for the whole simulation, representing acute deformation or disruption. This increased fluctuation in loops could be due to the (E100K) mutation. The average calculated values for the RMSF of the WT-SOD1, the E100K mutant, and the protein-ligand complexes associated with the metal-binding and electrostatic loops are presented in Table 4.

**TABLE 4:** The average value computed for root-mean-square fluctuation (RMSF) of WT-SOD1, E100K, and mutant protein-ligand complexes. The data were expressed as the mean ± SD (n = 3).

Average RMSF values	Loops	
	Metal binding loop	Electrostatic loop
WT-SOD1	0.244 ± 0.096	0.272 ± 0.09
E100K	0.321 ± 0.135	0.3 ± 0.11
E100K-Fisetin	0.292 ± 0.09	0.287 ± 0.11
E100K-Puerarin	0.295 ± 0.099	0.374 ± 0.129
E100K-Peonidin	0.31 ± 0.097	0.393 ± 0.14

The detected instabilities caused by the situation of loop IV close to the boundary of two monomers might crucially destabilize the dimeric nature of SOD1, which consecutively affects the folding process and enzymatic function. Significantly, mutations in SOD1 can potentially lead to misfolding and trigger its eventual aggregation. Loop VII has been shown to produce the best electrostatic field for absorbing superoxide anion radicals (Arnesano et al., 2004), and the distortion brought on by this substitution interferes with the orientation of the metal ligands for the dismutation reaction, dropping its catalytic performance. Additionally, the precise elasticity observed in pathogenic residues in loops might be a consequence of the folding’s conformational changes in the E100K mutant following interaction with ligands. Notably, Fisetin binding to E100K may be able to reduce the misfolding and pathogenic tendencies of this protein.

**FIGURE 8:**
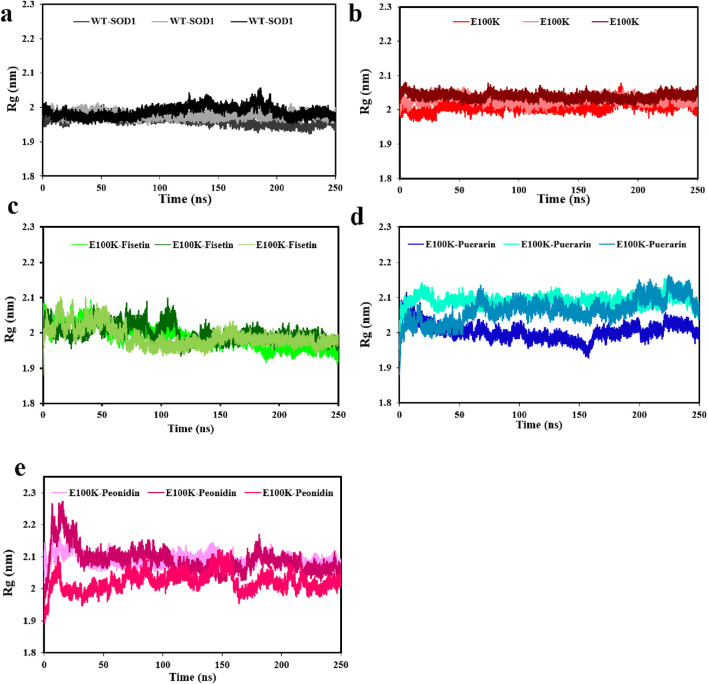
Radius of gyration (Rg) **(a)** WT-SOD1 and, **(b)** E100K upon interacting with flavonoids: **(c)** Fisetin, **(d)** Puerarin, and **(e)** Peonidin. Rg values ​​ were performed from three independent trajectories for each WT, mutant and protein-ligand complexes. Data were expressed as mean ± SD (n = 3).

The relative compactness of the protein structure, which is connected to the stability of the protein with the total of the intramolecular interactions in the protein structure, is shown by the radius of gyration (Rg) (Wise-Scira et al., 2013). The Rg measures the root-mean-square distance of the atom’s motion from its center of mass, which was investigated for E100K and protein-flavonoid complexes. Averagely, calculated Rg corresponding with WT-SOD1 (Figure 8a), and E100K mutant (Figure 8b) during the simulation was 1.97 ± 0.012 and 2.02 ± 0.011 nm, respectively, which decreased to 1.98 ± 0.027 nm due to Fisetin interaction with E100K (Figure 8c). However, the E100K mutant protein changed to 2.05 ± 0.023 and 2.06 ± 0.028 nm due to interaction with Puerarin and Peonidin, respectively (Figures 8d,e). Although these changes were minor, they may affect the process of inhibiting protein aggregation. Remarkably, by inspecting their trajectories it is relatively superficial that none of them are coordinated with one another, although there is no substantial variance between the mean Rg values of the E100K mutant and the protein-ligand complexes. The pathologically distinct dynamic position of the E100K mutant is structurally altered in interaction with the flavonoids: Fisetin, Puerarin, and Peonidin, which was clearly evident based on the initial evaluation of MD simulation pathways due to structural perturbations caused by the mutation.

**FIGURE 9:**
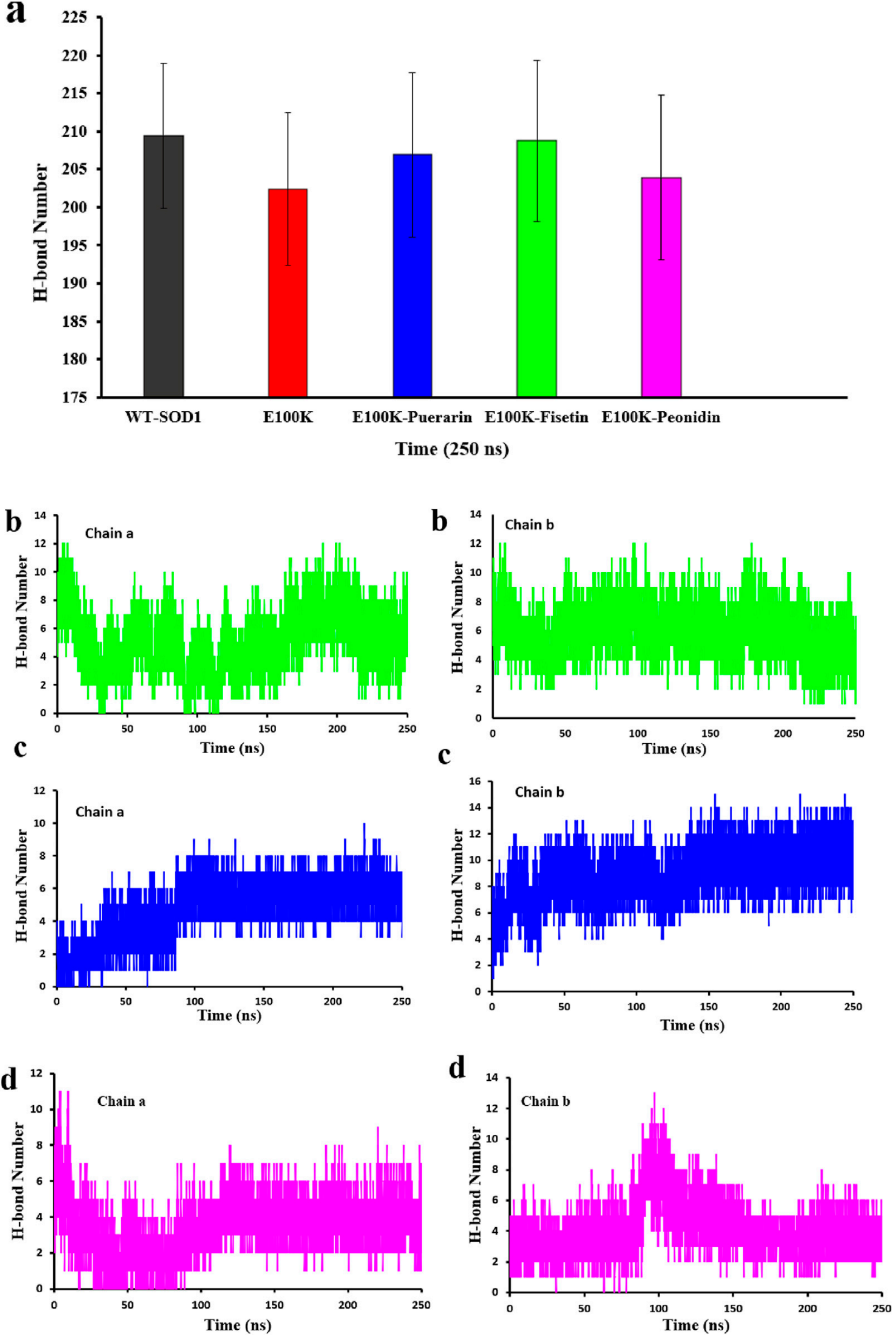
**(a)** Protein-protein hydrogen bond profiling of WT-SOD1 and E100K mutants following interaction with flavonoids: Puerarin, Fisetin, and Peonidin. Protein-ligand hydrogen bond profiling of the E100K mutant following interaction with flavonoids: **(b)** Fisetin, **(c)** Puerarin, and **(d)** Peonidin. H-bond values ​​ were performed from three independent trajectories for each WT, mutant and protein-ligand complexes. Data were expressed as mean ± SD (n = 3).

It is known that hydrogen bonds are the primary links that respond to structural perturbations produced by several mutations. Hydrogen interactions indicate bond firming and stability of any chemical structure, which is vital in the stability of protein structure (Alemasov et al., 2017; Khare and Dokholyan, 2006). Furthermore, it was reported in a study that the loss of the same residual site and gatekeeper amino acids, which have a negative charge, on the external part of protein pointedly declines the permanency of SOD1 and leads to aggregation (Tompa and Kadhirvel, 2020). Hence, we performed H-bonding analysis for WT-SOD1 and E100K and protein-ligand complexes (Figure 9a). On average, the hydrogen bond protein-protein changes for WT and E100K mutant proteins were 209 ± 9 and 202 ± 10 hydrogen bonds, respectively. By substituting Glu to Lys, the results showed that the mutation banned the development of hydrogen bonds and the number of H-bonds was significantly reduced, thus causing a decline in intermolecular interactions in the protein. While these variations are partial and local, they may affect the permanency of the structure and activity of the enzyme. Generally, 206 ± 10, 208 ± 10, and 203 ± 10 hydrogen bond protein-protein interactions were observed for protein-ligand complexes Puerarin, Fisetin, and Peonidin, respectively. Hence, the outcomes confirmed that the designated flavonoid molecules increase the number of hydrogen interactions in the mutant-ligand complexes, analogous to the outline in WT-SOD1. Besides, the outcomes proposed that Fisetin was the unsurpassed applicant for reestablishing the H-bonding outline, followed by Puerarin.

However, we also computed the protein-ligand hydrogen bond for both SOD1 chains in order to have a better understanding of the ligand’s impact on the mutant structure. The development and stability of inhibitor-enzyme complexes are significantly influenced by the direction and degree of hydrogen bonding. Additionally, as a measure of the stability of these connections, Figures 9b–d displays changes in the average number of H-bonds between replicas. Interestingly, Fisetin’s hydrogen bonds were more durable than those of other ligands, even though it had the lowest binding energy in comparison to Puerarin. This suggests that hydrophobic interactions and hydrogen bonds work together to prevent Fisetin from aggregating. The number of protein-ligand hydrogen bonds for Fisetin, Puerarin and Peonidin were 5 ± 1, 4 ± 1 and 3 ± 1 for chain (A) and for chain (B) 6 ± 2,4 ± 1 and 4 ± 1, respectively. These results are consistent with molecular docking (Figures 2, 3). Hypotheses on the correlation between patient survival time and the stabilizing and destabilizing effects of mutations in various structural and functional areas of SOD1 were made feasible by the analysis. To further understand the mechanisms of the hydrogen bonding influence on the development of pathogenic features of SOD1 mutations, more study employing potent molecular modeling methods is obviously required. As already stated (Srinivasan and Rajasekaran, 2017; Alemasov et al., 2022).

**FIGURE 10:**
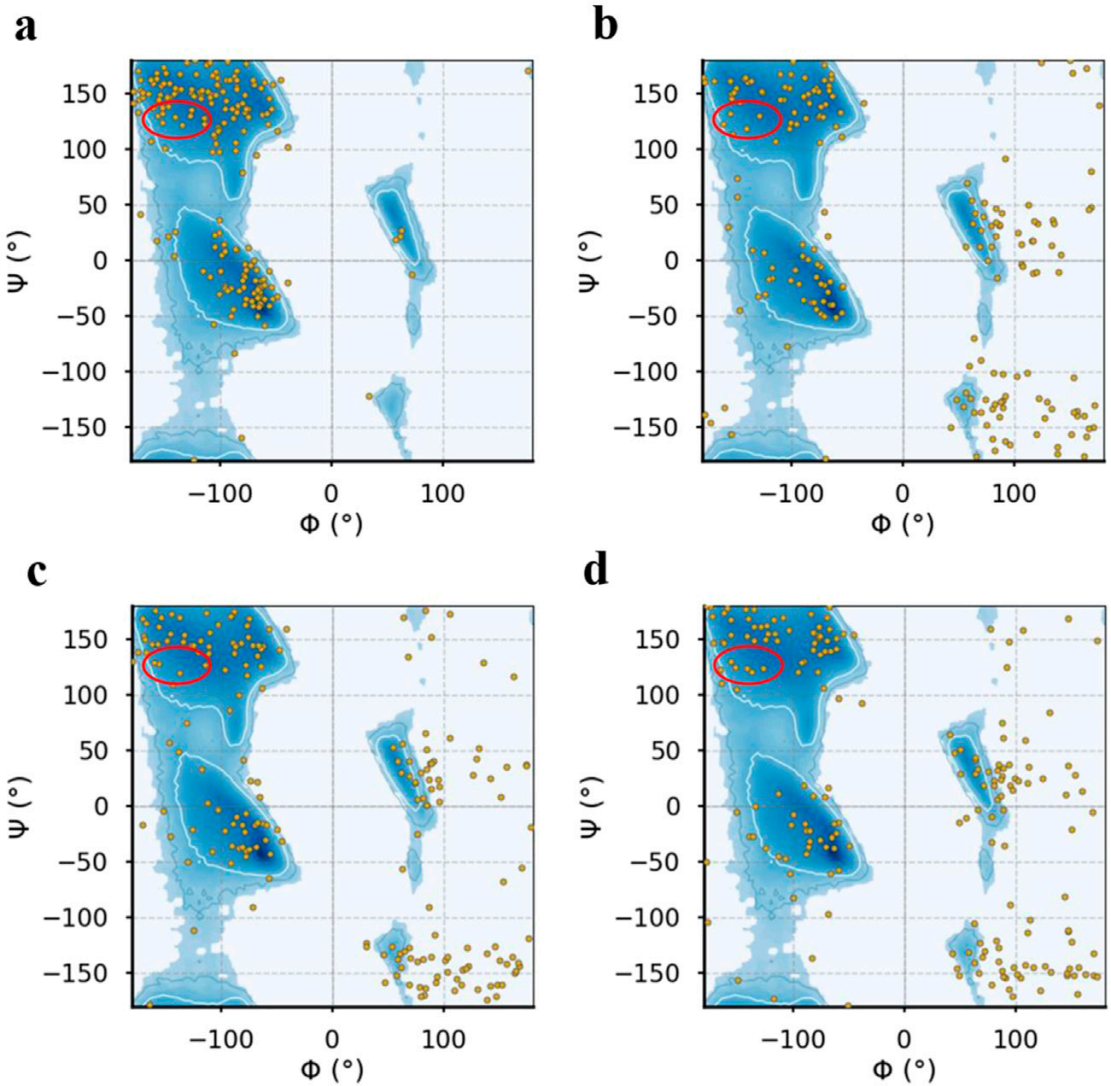
Represents the Ramachandran plot for the **(a)** E100K mutant alone and **(b)** in interaction with flavonoids: Fisetin, **(c)** Puerarin and **(d)** Peonidin.

Concomitantly, it is important to know that a specific pathological feature of neurological illnesses associated with ALS caused by genomic alterations and other unclear biomolecular factors is the transformation of the secondary structure of the pathogenic protein into β-sheet-rich amyloid fibrils (Milner-White et al., 2006). Hence, it is enough to say that the driving factor in supporting protopathic protein amyloid formulation tendencies is a dynamic increase in β-sheet composition, and preventing the formation of β-sheets can effectively disrupt amyloid formulation and lead to a decrease in pathology. As previously reported, mutations in SOD1 can cause the creation of amyloid fibrils and aggregates in the protein (Ivanova et al., 2014). Investigational studies also stated that increasing the tendency of β-sheets in protein aggregates usually prevents them from being exposed to hydrophobic interactions (Chiti and Dobson, 2006). Accordingly, secondary conformational variations in the E100K were determined by DSSP (Dictionary of Secondary Structure in Proteins) to evaluate the inhibitory impacts of protein-ligand structure on SOD1 conformational transitions during simulation. The temporal evolution of the tendency in the secondary structure of mutant WT-SOD1, E100K, and mutant-ligand interactions is shown in Table 5. It can be concluded that the tendency of β-sheets in the E100K mutant (40%) is significantly increased compared to WT (35%). (Figure 10a). Furthermore, the turns in WT were more abundant (14%) than in the E100K mutant (10%), thus representing that the decrease in the content of turns led to the increase in the content of β-sheets (Dong et al., 2016). We detected a transformation in the secondary structure tendency as a result of binding the mutant with protein-ligand complexes, and the propensity of β-sheets in interaction with Fisetin was significantly reduced to 36%, which indicates that Fisetin can have an effect in restoring the structure (Figure 10b). Upon interaction with Puerarin, the composition of β-sheets E100K mutant decreased from 40% to 38% (Figure 10c). However, the β-sheet content for the E100K mutant complex with Peonidin was 39% (Figure 10d). From these results, it can be inferred that the biomolecular interaction of E100K-flavonoids changes the structural orientation of residues in E100K, which reduces the content of β-sheets. Furthermore, another significant result from this investigation was that after the interaction of the E100K protein with ligands, the content of alpha-helices was increased compared to the unbound mutant. Analysis of the secondary structure corroborates the idea that these flavonoid complexes employ analeptic ability in restoring the E100K mutant to its WT-SOD1 form because both types share the same residual site for helices (Strange et al., 2007). According to the results, it seems that Fisetin is more effective by reducing the β-sheet and preserving the alpha-helix compared to other flavonoids. Overall, the simulation results provide an implication regarding the efficacy of flavonoids on mutant SOD1, which restored the variations in the secondary structure foundations caused by mutation compared to what was observed in the WT structure. Hence, it is suggested that mutant-ligand complexes (polyphenolic flavonoids) can help restore the WT-like secondary structure foundations and diminish the protein aggregation caused by the E100K mutant.

**TABLE 5:** Secondary structural analysis related to WT-SOD1, E100K and its flavonoid-based compounds. The data were expressed as the mean ± SD (n = 3).

System	Secondary structure elements (%)					
	Coil	β-Sheet	β-Bridge	Bend	Turn	α-Helix
WT-SOD1	27±0.5	**35**±1	4±1	15±0.5	14±0.5	5±1
E100K	29±2	**40**±2	2±0.5	18 ± 2.5	10±1	2±0.5
E100K-Fisetin	28±0.5	**36**±0.5	3±1	16±1	13±2	4±0.5
E100K-Puerarin	26±2	**38**±1	3±1	17±2	12±1.5	4±1
E100K-Peonidin	25±1	**39**±1	3±0.5	18±1	11±1	4±0.5

### 3.7 Essential dynamics

**FIGURE 11:**
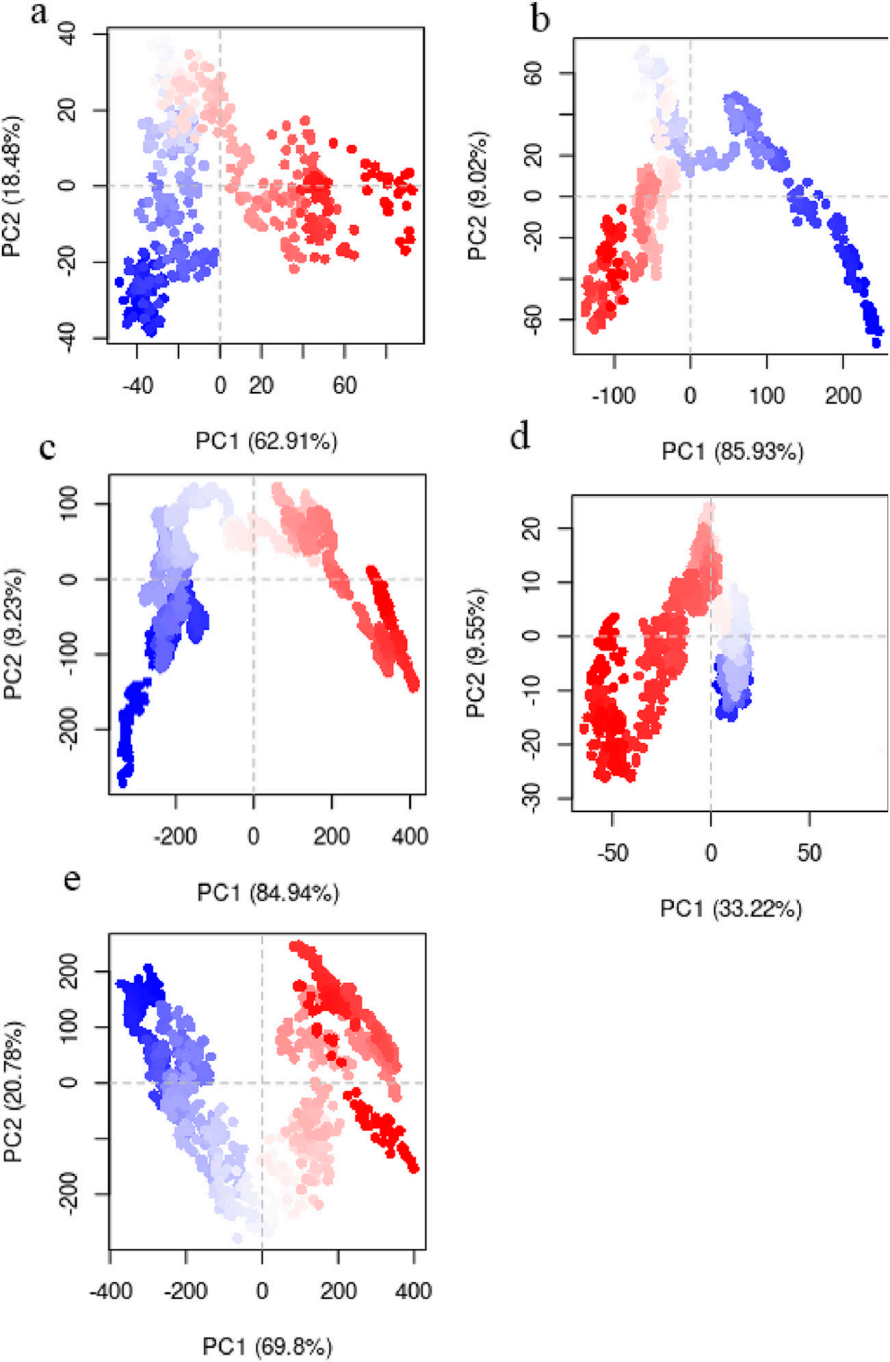
Principal component analysis (PCA) obtained by studying routes of **(a)** WT-SOD1, **(b)** Variations in mutant E100K proteins upon its interaction with flavonoids: **(c)** Fisetin, **(d)** Puerarin and **(e)** Peonidin.

Essential dynamics (ED) is another term for PCA when it comes to analyzing MD simulations (Pereira et al., 2020). Protein dynamics may be described with fewer dimensions thanks to ED linear, which converts the high-dimensional and complicated data found in a molecular trajectory into a low-dimensional space where the large-scale protein movements take place. A covariance matrix built with the Cartesian coordinates that represent atomic locations is used in this statistical approach to gradually filter the observed changes in a molecule’s route from the biggest to the lowest spatial-scale (David and Jacobs, 2014). The necessary protein movements may be separated from the others thanks to ED. The remaining motions reflect minor, inconsequential local changes, but the essential motions (i.e., the largest-scale motions) are often physiologically relevant movements like opening, shutting, and flexing. The first two PCA modes (principal components) frequently include the important movements (Stein et al., 2006). We conducted PCA on the Cα atoms of the protein, as these atoms provide a good representation of the overall conformational changes. PC1 and PC2 characterized 81.3%, 94.9%, 94.17%, 42.77% and 90.58% of system motility for WT-SOD1 and its variants E100K, protein-ligand complexes (Fisetin, Puerarin, and Puerarin), respectively, indicating that they encompass the major conformational motions (Figure 11). The projections of the trajectories onto the first two principal components (PC1 and PC2) are shown in the figure below. These plots reveal distinct clusters corresponding to different conformational states and transitions between them. It appears that the simulations sampled several transitions between the conformational states based on the continuous distribution of points along the PCs. The immediate conformations’ hue shifted from blue to red with time, according to the PCA scatter plots. The subsequent color changes (from blue to white to red) in the direction of PC1 showed that the conformation in both systems progressively changed to a different state during the experiment. As was previously mentioned, the PCA diagram’s point distribution shows a gradual shift in hue from blue to red. Put another way, the color shift in the direction of PC1 from red to white and from white to red indicates that the conformation has progressively changed states during the simulation.

### 3.8 Free energy landscape

**FIGURE 12:**
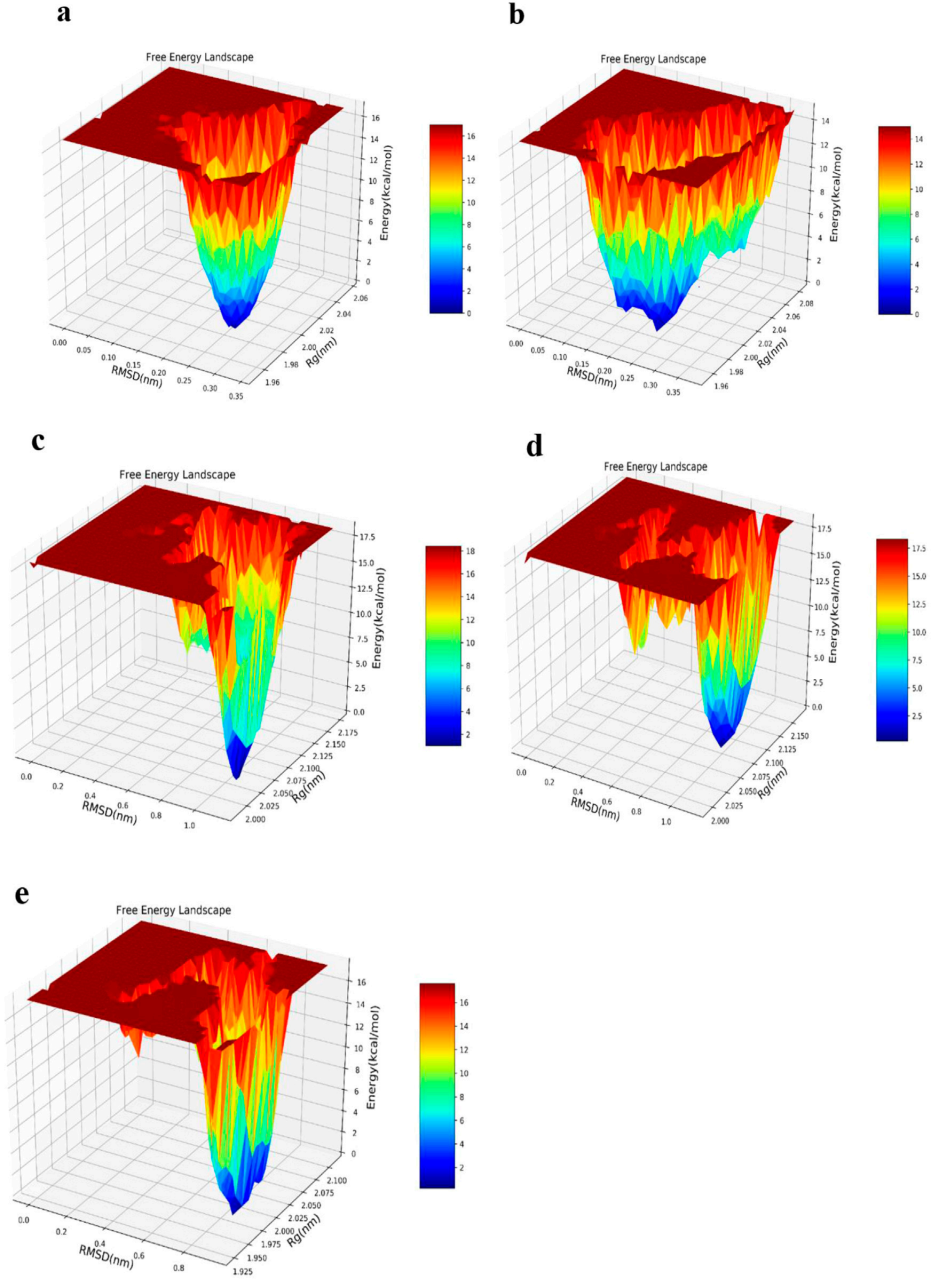
Free energy landscape (FEL) obtained by studying routes of **(a)** WT-SOD1, **(b)** Variations in mutant E100K proteins upon its interaction with flavonoids: **(c)** Fisetin, **(d)** Puerarin and **(e)** Peonidin. The FEL was designed using Rg and RMSD as its reaction coordinates, with energy values displayed along the Z-axis, as indicated by the color bar.

Aggregation of protein is investigable by considering the various conformational statuses engaged in the free energy landscape (FEL) (Zheng et al., 2013). Amyloid development mainly occurs as a result of misfolding in protopathic proteins, which ultimately transform into fibrils (Adamcik and Mezzenga, 2018). Accordingly, the FEL was assessed for the WT-SOD1 protein, E100K mutant, and the ligand-protein complexes. Similarly, an FEL was created to compute the frequency of global energy minima in order to investigate the pathology of the E100K mutant during the simulation time period (Yang et al., 2013). FEL analysis used RMSD and Rg as coordinates to show the compaction and structural steadiness of the protein during the molecular dynamics period. The Gibbs free energy of the generated FEL of WT-SOD1 and the E100K mutant ranged from 0 to 20 kcal/mol, as shown in Figure 12. The free energy ranges from blue to red, challenging the blue region for the minimum global energy composition and the red for metastable states. We concluded that the substitution mutation in SOD1 significantly transformed the folding pattern, possibly leading to increased RMSD values toward the multiple energy minimum achieved by the mutant conformers. The minimum global energy number for a native protein is usually to one. The free energy landscape for the WT-SOD1 structure shows a unique favorable region (Figure 12a) that lies between Rg and RMSD values of ∼1.97 nm and ∼0.26 nm, respectively. However, results for the E100K mutant protein indicated multiple global energy minima that may perhaps arise in various thermodynamically conceivable conformational statuses, which might cause it to misfold and ultimately lead to amyloid. The effect of substitution polymorphisms on this progression led to the creation of several promising free energy basins (Figure 12b), which were 1.98 and 0.23 nm for Rg and RMSD, respectively. Therefore, as the free energy profile showed, Glu100 present in WT gives rise to the occurrence of the limited free energy basin throughout RMSD and Rg, which by replacing Lys led to the change of SOD1 proteins and reaching several global free energy minima. Furthermore, the free energy domain obtained by WT-SOD1 was lower than the conformational structures of the E100K mutant, indicating a more favorable conformation adopted by the WT conformers than the mutant. Altogether, the results support the idea that an augmented relative ratio of various conformations with smaller free energy in either form of the protein leads to the predominance of unfolded states. Furthermore, in confirmation of our results indirectly compared to the results of previous studies, it suggests that the aggregated proteins obtain the minimum multiple energy for the conformational structures that indicate the development of toxic aggregates in mutant SOD1 (Keerthana and Kolandaivel, 2015; Viet et al., 2011). Moreover, the FEL was investigated for conformers of mutant-ligand complexes through the coordination of RMSD and Rg. Henceforth, the E100K mutant has a global energy minimum after binding with Fisetin that can be found at Rg ∼2 nm and RMSD ∼0.1 nm, due to the Fisetin effect on mutant SOD1 that adjusts the compaction and conformational firmness of the compounds (Figure 12c). The incidence of a minimum global peak defends the theory that the active contact of Fisetin with mutant SOD1 surges conformational permanency and reduces compaction. Hence, it thermodynamically and structurally controls the mutagenic state through a distinct SOD1 pathway. Moreover, the FEL of the E100K complex in interaction with Puerarin and Peonidin in global minimum free energy basins (Figures 12d,e) varied between Rg values of (2.1 and 2 nm) and RMSD values of (0.9 and 0.8 nm), respectively. The structural diversity of polyphenolic flavonoid composites (Puerarin and Peonidin) in mutant SOD1 has different effects on structural changes. Finally, the results from the study of molecular interactions, structural firmness, and secondary structure orientation, along with the free energy perspective confirmed the inhibitory effect of Fisetin, unlike other flavonoids, on mutant SOD1 in comparison with that of WT-SOD1, as previously reported. (Qassim et al., 2023).

### 3.9 Cross-correlation matrix

**FIGURE 13:**
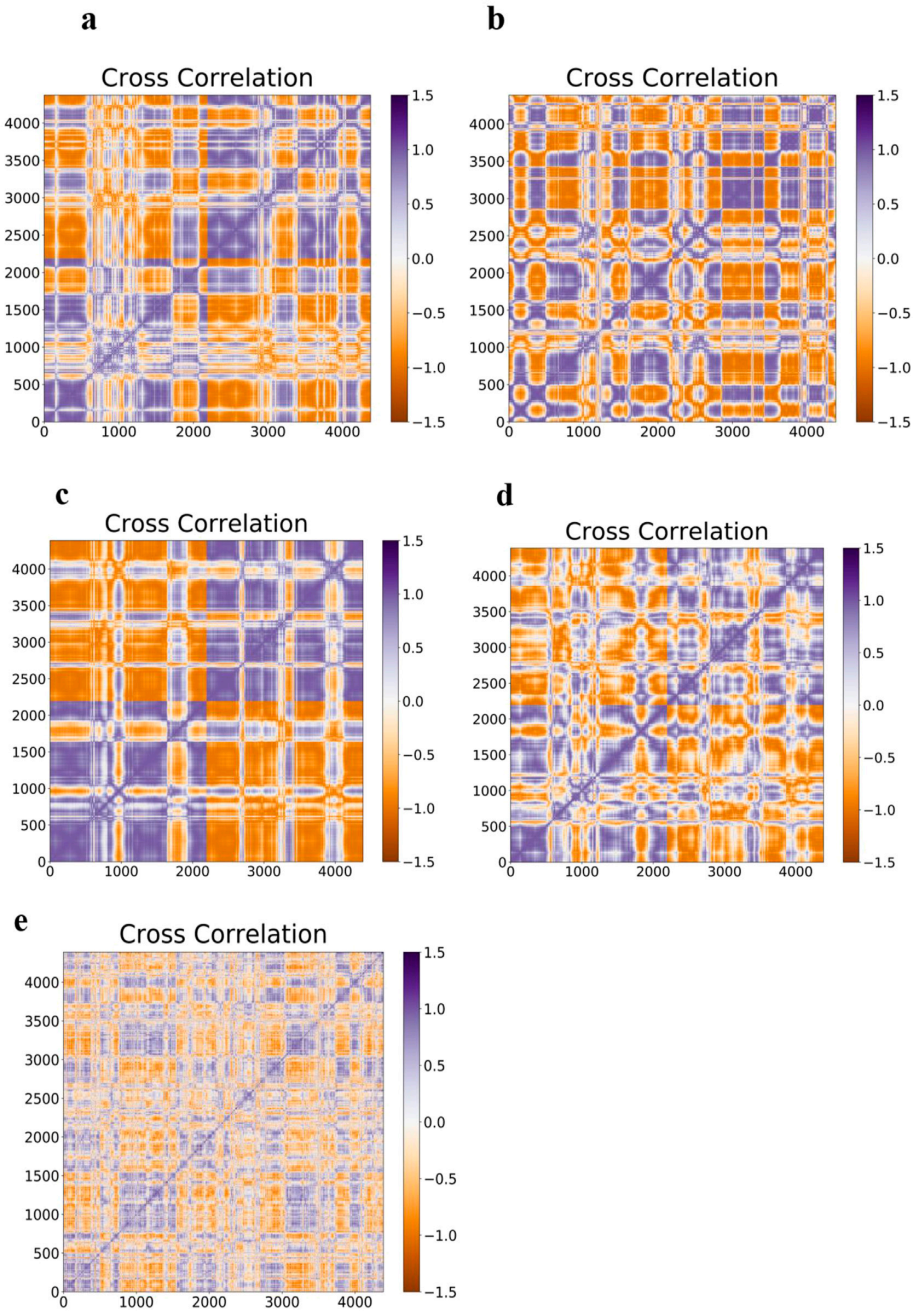
It illustrates the Covariance matrix related to the axial atoms for; **(a)** WT-SOD1, **(b)** Interatomic Covariance matrix disparities in altered E100K SOD1 proteins upon its contact with flavonoids: **(c)** Fisetin **(d)** Puerarin, and **(e)** Peonidin.

Furthermore, a noticeable form of interatomic correlation motions that could give rise to its pathogenicity was provided by molecular simulation studies of amyloid-β folding (Yang and Teplow, 2008). For this purpose, the interatomic motion covariance of E100K mutation and the intervention of flavonoid compounds due to the biochemical effect were calculated. Therefore, larger cross-correlation values indicate more correlation or anti-correlation between two residues. Overall, the interatomic cross-correlation analysis indicated that the mutation had significantly changed the protein’s internal motion and flexibility due to the disruption in the SOD1’s intermolecular connection. A cross-correlation value equal to zero implies accidental residual movements with no correlation. The results (Figure 11) demonstrated unequivocally that the mutant amyloidogenic protein’s covariance pattern of unique pathogenic interatomic motion was considerably altered as a result of binding with flavonoids. It demonstrates how the E100K mutant is affected by flavonoids, Fisetin, Puerarin, and Peonidin to lessen its disease. Strangely, the mutant exhibits a lower degree of correlation between certain residues than the WT (Figures 13a,b). Conversely, the total motion correlation rose when mutation was introduced. All mutant systems displayed more overall motion correlation in catalytic areas, which is consistent with RMSF analysis. The WT system showed less anti-correlated movements and more inter/intra-correlated motions. The stability of dimer formation may be weakened by the significant anti-correlated movements that have been seen in the mutant systems. Remarkably, the number of atoms in the mutant that had substantially connected motion decreased when Fisetin was added (Figure 13c). On the other hand, compared to other substances, the anticorrelation movements found in the altered residues with Puerarin binding were less obvious (Figure 13d). Moreover, the interatomic cross-correlation matrix demonstrated that upon binding to Peonidin, the mutant’s robust intermolecular interactions could not be distinguished from those of other substances (Figure 13e). Overall, Fisetin is more effective than other drugs at modifying the stability and flexibility of mutant SOD1 mobility, as demonstrated by the relevance of the dynamic interatomic cross-correlation matrix.

### 3.10 In silico drug ADMET evaluation

The process of creating new medications is harder, more expensive, riskier, and less successful every time. For medications to be successful, pharmacokinetics, toxicity, and potency must interact. The creation of computer techniques to maximize toxicity and pharmacokinetic characteristics may speed up and improve the process of finding new medication candidates. Predicting ADMET-related aspects of novel medications is challenging since there are few relationships between various physicochemical qualities and pharmacokinetic and toxicological properties. New techniques for understanding, examining, and predicting the ADMET (Absorption, Distribution, Metabolism, Excretion, Toxicity) properties of tiny compounds are currently needed in order to improve compound quality and success rate (Cumming et al., 2013). The logical development of anti-ALS medications depends on a drug’s pharmacokinetic characteristics. As a result, the test chemicals’ toxicity was assessed *in silico*. Table 6 presents the findings. Three compounds were examined utilizing the pKCSM technique in ADMET screening. The *Salmonella typhimurium*/reverse mutation assay (AMES assay), LD50, and hepatotoxicity prediction serve as the foundation for the toxicity potential parameter (Fourches et al., 2010). Based on the anticipated outcomes, puerarin may be a hERG II inhibitor. It was also anticipated what the human maximum acceptable dosage would be. These findings align with the ADMET characteristics of flavonoids that have been previously investigated to comprehend their pharmacokinetic profile (Arsito et al., 2023).

**TABLE 6:** Prediction of toxicity parameters of flavonoids (pKCSM program).

Parameter		Fisetin	Puerarin	Peonidin	Unit
Absorption	Water solubility	−3.181	−2.72	−3.058	(log mol/L)
	Caco2 permeability	0.058	0.223	−0.133	(log Papp in 10 ^-6^ cm/s)
	Intestinal absorption (human)	83.752	67.446	89.163	(% Absorbed)
	Skin Permeability	−2.735	−2.735	−2.735	(log Kp)
	P-glycoprotein substrate	Yes	Yes	Yes	Categorical
	P-glycoprotein I inhibitor	No	No	No	Categorical
	P-glycoprotein II inhibitor	No	No	No	Categorical
Distribution	VDss (human)	0.718	0.377	0.563	(log L/kg)
	Fraction unbound (human)	0.166	0.187	0.109	(Fu)
	BBB permeability	−1.039	−1.204	−1.256	Categorical
	CNS permeability	−2.282	−3.594	−2.297	(log PS)
Metabolism	CYP2D6 substrate	No	No	No	Categorical
	CYP3A4 substrate	No	No	Yes	Categorical
	CYP1A2 inhibitior	Yes	No	Yes	Categorical
	CYP2C19 inhibitior	No	No	No	Categorical
	CYP2C9 inhibitior	Yes	No	Yes	Categorical
	CYP2D6 inhibitior	No	No	No	Categorical
	CYP3A4 inhibitior	No	No	No	Categorical
Excretion	Total Clearance	0.421	−0.007	0.633	(log ml/min/kg)
	Renal OCT2 substrate	No	No	No	Categorical
Toxicity	AMES toxicity	No	No	No	Categorical
	Max. tolerated dose (human)	0.579	0.642	0.568	(Log mg/kg/day)
	hERG I inhibitor	No	No	No	Categorical
	hERG II inhibitor	No	Yes	No	Categorical
	Oral Rat Acute Toxicity (LD50)	2.465	2.641	2.408	(mol/kg)
	Oral Rat Chronic Toxicity (LOAEL)	1.921	4.85	2.434	(Log mg/kg_bw/day)
	Hepatotoxicity	No	No	No	Categorical
	Skin Sensitization	No	No	No	Categorical
	*T.Pyriformis* toxicity	0.376	0.285	0.319	(Log ug/L)
	Minnow toxicity	2.273	4.188	1.409	(Log mM)

Choosing which flavonoids—Fisetin, Puerarin, and Peonidin—have the most medicinal capacity for reducing the pathogenic effects of the E100K mutation is now our most urgent task. Fisetin, out of the three flavonoids, has the maximum result in reducing the aggregation and amyloidogenic characteristics of the E100K mutant. Our results are in alliance with previous studies on the competence of binding flavonoids with the E100K mutant, which confirm Fisetin’s potential impact on transformed β-sheets and FEL. Our research also exposed that Fisetin, a powerful polyphenolic flavonoid that ordinarily promotes amyloidogenic proteins including those engaged in Alzheimer’s disease, amyloid beta, and α-synuclein, may be one of the flavonoids most useful for minimizing the consequences of E100K mutant amyloid (Ahmad et al., 2017; Samanta et al., 2022). Fisetin also crosses the blood-brain barrier and its concentration in the central nervous system reaches its peak 2 h after injection (Gelderblom et al., 2012). Contrarily, prior findings indicate that Peonidin and Puerarin have a long history of usage in controlling cancer, endometriosis, Parkinson’s disease, Alzheimer’s disease (Liu et al., 2023), diabetes, and its consequences, osteonecrosis, cardiovascular and cerebrovascular illnesses (Zhou et al., 2014), while our results indicated that these compounds as the utmost operative flavonoid in alleviating the amyloid impacts of mutant SOD1 were not particularly effective. Also, the lack of experimental analysis in corroborating flavonoid’s potency should be addressed. Accordingly, one must delve into reports from prior pathogenic SOD1-flavonoids research, which indicates a strong correlation and affirmation between theoretical-computation model of flavonoids’ potency against aberrant SOD1 and experimental findings, which ratifies said flavonoids’ analeptic potential in mitigating pathogenic behavior of SOD1 (Srinivasan and Rajasekaran, 2018a; Srinivasan and Rajasekaran, 2016; Srinivasan and Rajasekaran, 2017; Srinivasan and Rajasekaran, 2018b). Next, we will discuss the physicochemical and pharmacological properties of Fisetin.

#### 3.10.1 Chemistry

Fisetin (3,3′,4′,7-tetrahydroxyflavone) is a naturally occurring yellow pigment categorized as a polyphenolic compound within the flavonoid subclass. It is found in small to moderate amounts (0.1–160 μg/g) across a variety of fruits and vegetables, including strawberries, apples, persimmons, lotus roots, onions, grapes, kiwis, peaches, tomatoes, and cucumbers. Notably, strawberries contain the highest known concentration of Fisetin, reaching up to 160 μg/g (Arai et al., 2000). Structurally, Fisetin is a flavonol characterized by a diphenylpropane backbone, consisting of two aromatic rings connected by an oxygen-containing heterocyclic ring. The molecule includes four hydroxyl (OH) groups and one carbonyl (oxo) group (Jash and Mondal, 2014). The compound’s bioactive properties are largely attributed to specific functional groups, notably the hydroxyl groups located at the 3, 7, 3′, and 4′positions, the carbonyl group at position 4, and a double bond between carbon atoms C2 and C3. This structural configuration—especially the C2 = C3 bond, and hydroxyls at C-7, C-3′, and C-3—contributes significantly to Fisetin’s antioxidant capacity (Sengupta et al., 2004). Despite its promising pharmacological attributes, Fisetin faces several challenges, including low systemic availability (44.1%), limited solubility in water (10.45 μg/mL), and high lipid solubility (logP of 3.2) (Mehta et al., 2018). Pharmacokinetic studies have shown that Fisetin is rapidly absorbed and metabolized via phase II reactions, resulting in the formation of sulfate and glucuronide conjugates. Following intraperitoneal (i.p.) administration at a dose of 223 mg/kg, peak plasma concentrations (2.53 μg/mL) were observed within 15 min (Shia et al., 2009). This rapid clearance necessitates repeated dosing to maintain therapeutic levels. Improving bioavailability is therefore critical—not only to reduce dosing frequency but also to minimize potential adverse effects. Interestingly, studies on animal models have demonstrated that Fisetin can cross the blood-brain barrier efficiently and accumulates within brain tissue following both oral and i. p. routes of administration (Krasieva et al., 2015). Since Fisetin is being explored for its neuroprotective potential, effective brain delivery is a key requirement for its therapeutic success. Given these pharmacokinetic limitations, strategies to enhance its bioavailability are of great importance. Bioavailability, as a determinant of bioefficacy, plays a pivotal role in achieving therapeutic outcomes; insufficient systemic levels hinder biological responses. Research suggests that co-crystallization with compounds like caffeine, isonicotinamide, and nicotinamide can improve both the solubility and oral absorption of Fisetin (Sowa et al., 2014). Additionally, forming inclusion complexes with cyclodextrins and employing nanoencapsulation techniques (Cai et al., 2022) have shown promise in enhancing both solubility and pharmacodynamic effects. Thus, chemical modification of its structure and the use of advanced drug delivery systems such as nanocarriers present viable approaches to unlocking Fisetin’s full therapeutic potential. The following sections will explore current and emerging strategies aimed at improving its bioavailability.

#### 3.10.2 Nanoparticle-based delivery systems (polymeric nanoparticles)

Nanoparticles possess a high surface-to-volume ratio, which significantly enhances the solubility of bioactive substances. Incorporation of Fisetin into polymeric nanoparticles has demonstrated anti-cancer properties. In a study by Feng et al., Fisetin was successfully encapsulated within poly (lactic acid) (PLA) through the spontaneous emulsification-solvent diffusion method (Feng et al., 2019). Additionally, conjugation with monomethyl poly (ethylene glycol)-poly (ε-caprolactone) (MPEG-PCL) has been explored as a viable strategy to further increase Fisetin’s bioefficacy (Yang et al., 2014).

#### 3.10.3 Human serum albumin nanoparticles

Among various polymeric drug delivery platforms, human serum albumin nanoparticles (HSA-NPs) stand out due to their excellent biocompatibility, biodegradability, and tolerability upon administration. These properties make them promising carriers for pharmaceutical applications (Elzoghby et al., 2012).

#### 3.10.4 Nanoemulsions

Nanoemulsions are finely dispersed systems comprising two immiscible liquids, such as oil and water, stabilized by surfactants or co-surfactants to produce a homogenous phase. These dispersions, typically within the 20–200 nm size range, can enhance solubility and absorption of lipophilic drugs (Solans et al., 2005). A newer form, known as self-nanoemulsifying drug delivery systems (SNEDDS), comprises a mixture of oil, surfactants, co-surfactants, and the active drug, forming a nanoemulsion upon exposure to gastrointestinal fluids. This spontaneous emulsification boosts surface area and promotes rapid absorption, improving oral bioavailability (Kuruvila et al., 2017). SNEDDS incorporating Fisetin have demonstrated improved solubility, permeability, and therapeutic efficacy.

#### 3.10.5 Lipid structures (solid lipid nanoparticles)

First developed in the 1990s, solid lipid nanoparticles (SLNs) were introduced as alternatives to liposomes and polymeric systems to address limitations of earlier carriers (Wissing et al., 2004). These carriers, generally <1,000 nm in size, consist of lipids that remain solid at both room and physiological temperatures. Prepared typically via high-pressure homogenization, SLNs offer a protective and controlled-release environment for labile drugs. The use of natural, non-toxic lipids and avoidance of organic solvents contribute to their safety profile. Given their affinity for lipophilic molecules, SLNs are particularly suitable for enhancing the delivery and efficacy of compounds like Fisetin (Wissing et al., 2004). Considering this benefit, the researchers chose SLN as a carrier for Fisetin to improve its biological effect.

#### 3.10.6 Spherulites

Spherulites are a specialized form of multilamellar liposomes exhibiting a concentric, onion-like lamellar structure. Generated through mechanical shearing, they are composed of lecithin, cholesterol, polyethylene glycol, and other surfactants. Their unique structure offers enhanced stability compared to traditional liposomes, while retaining favorable delivery characteristics (Crauste-Manciet et al., 2015).

#### 3.10.7 Nanocrystals

Nanocrystals are composed entirely of the active pharmaceutical ingredient in crystalline form, without the use of carriers. These drug crystals, typically <1 µm in size, are coated with stabilizing agents to maintain suspension stability. Techniques such as milling, precipitation, and spray-drying are used to produce these particles (Junghanns and Müller, 2008). Nanocrystallization dramatically increases surface area and dissolution rate, improving solubility and intestinal absorption. This innovative formulation method has already found practical applications in pharmaceutical development (Fernandes et al., 2018).

#### 3.10.8 Complex with cyclodextrin

Cyclodextrins (CDs) are ring-shaped oligosaccharides composed of glucose units linked via α-1,4-glycosidic bonds, forming a cone-like structure. The hydrophobic internal cavity and hydrophilic exterior enable them to encapsulate nonpolar compounds, improving their solubility and stability. Among the most common types are α-, β-, and γ-CDs, containing six, seven, and eight glucose units, respectively (Saenger et al., 1998). Complexation with CDs has been proven effective in increasing the solubility and oral availability of hydrophobic compounds like Fisetin, offering a simple yet powerful strategy for enhancing bioavailability.

#### 3.10.9 Pharmacokinetic profile of fisetin

Several studies have examined the pharmacokinetics of Fisetin. Following intraperitoneal injection at 223 mg/kg in mice, a maximum plasma concentration of 2.5 μg/mL was observed within 15 min. The plasma levels declined in a biphasic pattern, with a short half-life of 0.09 h. Three metabolites were identified, including two glucuronide conjugates and a methoxylated derivative known as 3,4′,7-trihydroxy-3′-methoxyflavone (geraldol) (Touil et al., 2011). Geraldol was found to be the primary metabolite and rapidly formed after both intravenous (2 mg/kg) and oral (100–200 mg/kg) administration in mice (Jo et al., 2016). The oral bioavailability of Fisetin was reported to be 7.8% at 100 mg/kg and 31.7% at 200 mg/kg. Interestingly, geraldol showed higher Cmax and AUC values compared to the parent compound (Jo et al., 2016). Moreover, Fisetin and its conjugates are partially excreted via the bile through P-glycoprotein-mediated transport, which may be influenced by structural features such as the 3-hydroxyl group and the C2 = C3 double bond (Huang et al., 2018). In murine models, Fisetin was shown to be rapidly absorbed and extensively metabolized via glucuronidation and sulfation, with the highest concentrations observed in the kidneys, intestines, and liver (Shia et al., 2009). Significantly, studies have confirmed that orally administered Fisetin can cross the BBB, accumulating within brain tissues (Krasieva et al., 2015; Maher, 2009). Fisetin exhibited a high brain uptake potential *in vitro* (Maher, 2009); also, it was shown to disperse into the brain parenchyma after oral administration in mice (Krasieva et al., 2015). More, in addition to its BBB permeability, Fisetin can also affect hippocampal synaptic plasticity indirectly through the peripheral system (He et al., 2018). Results of a study showed that oral supplementation of Fisetin (9 mg/kg/day) in SOD1-G93A transgenic mice at 2 months of age improved motor function by delaying motor deficits. In addition, Fisetin treatment significantly increased the number of motor neurons in the spinal cord. This flavonoid also limited the progression of ALS and increased survival (Wang et al., 2018). Fisetin scavenges ROS and limits oxidative stress. It also increases the expression of SOD, catalase, GPx, HO-1. Numerous studies show that Fisetin is effective against AD, PD, HD, MS and ALS (Hassan et al., 2022). The multifaceted actions of Fisetin increase its potential for use in the prevention of neurodegenerative disorders. Finally, it can be said that extensive research on the use of Fisetin against CNS disorders and appropriate clinical trials provide the potential to combat neurological disorders. Despite its promising pharmacological profile, the low systemic availability of phytonutrients like Fisetin remains a barrier to clinical application. Therefore, a thorough understanding of its pharmacokinetics is essential for optimizing its therapeutic potential in humans (Rein et al., 2013). We hypothesized that Fisetin would be a practical treatment choice for dropping the adverse properties of E100K in light of the aforementioned association and consistent analytical results. Our findings may also help researchers to develop Fisetin-based medicines for E100K to lessen ALS. In summary, studies have shown that Fisetin provides various health benefits. Novel formulations should help to increase its bioavailability, which can then improve its therapeutic efficacy in particular diseases.

## 4 Conclusion

Protein misfolding and its buildup in fibrillar deposits are frequent characteristics of degenerative disorders. A *particular* example of neurodegenerative disease is ALS. Here, we describe the impact of SOD1 mutation, which causes protein instability and aggregation, ultimately leading to ALS. However, there has not been a specific medication product developed to stop ALS yet. Because they promise substantial medicinal benefits in contradiction of a diversity of neurodegenerative illnesses in individuals and because the majority of them also function as antioxidants and amyloid aggregation inhibitors, phenolic small molecule inhibitors are among the most significant natural substances. Natural substances are less harmful than synthesized chemical molecules, which can be a game-changer in the exploration for blockers of protein misfolding which will help with treating several fatal disorders. In a positive development, recent improvements in medication discovery were a consequence of testing the anti-amyloidogenic potential of flavonoids on protopathic peptides, such as SOD1. The E100K mutant’s tendency for protein aggregation was successfully and considerably reduced or prevented using a very effective flavonoid-based treatment candidate, which was discovered in this work using a rigorous computational technique. Even though Fisetin’s analeptic ability was not tested in this work, the results of protein-ligand studies and multiple pathway modeling data suggested that Fisetin may be a suitable therapeutic option for the treatment of E100K mutation. Last but not least, this result supported the notion of aggregation inhibition well and showed that Fisetin has a higher binding affinity than other flavonoids and has an inhibitory impact on mutant aggregation. Therefore, we can suggest that Fisetin may be used as a key element in the development of small molecule inhibitors of mutant SOD1 proteins. Hence, the results of this research indicated that a breakthrough in drug development based on the structure and design of anti-aggregation drugs may be made for the treatment of irreversible ALS afflicting humans.

## Data Availability

The raw data supporting the conclusions of this article will be made available by the authors, without undue reservation.
